# The relationship between the price and demand of alcohol, tobacco, unhealthy food, sugar-sweetened beverages, and gambling: an umbrella review of systematic reviews

**DOI:** 10.1186/s12889-024-18599-3

**Published:** 2024-05-10

**Authors:** Robyn Burton, Casey Sharpe, Saloni Bhuptani, Mike Jecks, Clive Henn, Nicola Pearce-Smith, Sandy Knight, Marguerite Regan, Nick Sheron

**Affiliations:** 1 Department of Health and Social Care, Office for Health Improvement and Disparities, 39 Victoria Street, London, England; 2 Institute for Social Marketing and Health UK, University of Stirling, Stirling, FK9 4LA Scotland, UK; 3 UK Health Security Agency (UKHSA), 10 South Colonnade, Canary Wharf, London, England; 4 The Roger Williams Institute of Hepatology, Kings College London, London, England

**Keywords:** Tax, Price, Price elasticity of demand, Alcohol, Tobacco, Unhealthy food, Sugar sweetened beverages

## Abstract

**Background:**

The WHO highlight alcohol, tobacco, unhealthy food, and sugar-sweetened beverage (SSB) taxes as one of the most effective policies for preventing and reducing the burden of non-communicable diseases. This umbrella review aimed to identify and summarise evidence from systematic reviews that report the relationship between price and demand or price and disease/death for alcohol, tobacco, unhealthy food, and SSBs. Given the recent recognition as gambling as a public health problem, we also included gambling.

**Methods:**

The protocol for this umbrella review was pre-registered (PROSPERO CRD42023447429). Seven electronic databases were searched between 2000–2023. Eligible systematic reviews were those published in any country, including adults or children, and which quantitatively examined the relationship between alcohol, tobacco, gambling, unhealthy food, or SSB price/tax and demand (sales/consumption) or disease/death. Two researchers undertook screening, eligibility, data extraction, and risk of bias assessment using the ROBIS tool.

**Results:**

We identified 50 reviews from 5,185 records, of which 31 reported on unhealthy food or SSBs, nine reported on tobacco, nine on alcohol, and one on multiple outcomes (alcohol, tobacco, unhealthy food, and SSBs). We did not identify any reviews on gambling. Higher prices were consistently associated with lower demand, notwithstanding variation in the size of effect across commodities or populations. Reductions in demand were large enough to be considered meaningful for policy.

**Conclusions:**

Increases in the price of alcohol, tobacco, unhealthy food, and SSBs are consistently associated with decreases in demand. Moreover, increasing taxes can be expected to increase tax revenue. There may be potential in joining up approaches to taxation across the harm-causing commodities.

**Supplementary Information:**

The online version contains supplementary material available at 10.1186/s12889-024-18599-3.

## Background

The profile of the leading causes of disease and death has changed over time, with conditions caused by commercial determinants of health progressively replacing infectious diseases, particularly in the global north [1–3]. Alcohol consumption, smoking, and excess weight are among the top 10 risk factors for disability adjusted life years (DALYs) globally, accounting for almost a fifth of all DALYs and over a quarter of deaths in 2019 [3]. In high-income countries, this increases to over a quarter of all DALYs and almost a third of all deaths.

Although an individual might only have one risk factor of alcohol use, smoking, or excess weight, many of these risks co-occur. For example, smokers are around three times more likely to drink at risky levels and up to 1.6 times more likely to have a poor diet [4, 5]. Multiple risks also cluster among the most deprived [4], and are therefore an important consideration for health inequalities. Multiple risks have clinical implications. A recent meta-analysis identified that the risk of disease and death from multiple risks is large, and for some outcomes, synergistic [6]. For example, the combined effect of risky alcohol consumption and excess weight on liver disease/death is 1.6 times greater than the sum of each risk on its own (95% confidence interval [CI] = 1.3, 1.9). Synergistic interaction was also seen for co-occurring risky alcohol consumption and smoking and oral cancers, where the combined effect was 3.8 times greater than the sum of each risk on its own (95% CI = 2.6, 5.4). Clearly, there are behavioural and clinical synergies between alcohol, smoking, and excess weight, but importantly, there are also policy synergies.

Current public health approaches to tackle alcohol consumption, smoking, and excess weight are somewhat siloed, as reflected in the global strategies across these three risks which include actions on price, marketing, and availability for each commodity on its own, but makes no reference to joining up these approaches to tackle multiple risks together in a comprehensive approach [7]. Within governments, health departments often have separate teams and policy approaches for alcohol, tobacco, and excess weight, and in finance departments, duty on related commodities are usually decided on a case-by-case basis. Many of the civil society organisations, charities, and medical academics who perform a vital advocacy role with policymakers also operate in these silos. Nonetheless, the evidence for the most effective and cost-effective approaches to preventing and reducing harm are broadly the same for tobacco, alcohol, and excess weight: increasing product price, restricting marketing, and reducing availability [7]. It is no coincidence that these comprise three of the four P’s of the marketing mix, the fourth being product, because the problems are generated by the products of commercially mediated organisations [8]. A requirement to reduce health harm is frequently in direct opposition to the generation of shareholder value [9].

Similarities in policy approaches to prevent and reduce alcohol-, tobacco-, and obesity-related harm can be seen across those implemented in the UK. For example, tobacco is subject to an annual tax escalator [10], and a sugar-sweetened beverage (SSB) tax came into force in 2018 [11]. Alcohol had a tax escalator between the years of 2008 and 2013 during which time deaths caused entirely by alcohol consumption (the directly attributable mortality) reduced, only to increase again when the measure was repealed [12, 13]. There are potential merits in considering policy areas together. For example, in the USA, increases in cigarette tax were associated with decreases in alcohol consumption among smokers [14]. In a UK study, the price elasticity of demand (PED) for alcohol was estimated to be -0.56, which reduced to -0.26 when the price of food was included in the analysis [15]. This suggests that pricing policies for alcohol may be undermined either if retailers offset an increase in alcohol price by decreasing the price of food, or if consumers have more disposable income due to the reductions in food price which can be spent on alcohol. Although price increases in medium-sugar drinks in the UK were associated with reductions in alcohol purchasing, increases in the price of high-sugar drinks were associated with an increase in purchasing of lager [16]. Finally, Australian modelling suggests that increasing alcohol tax is the most effective and cost-effective action to reduce obesity [17].

There is coherent logic in combining policy approaches for alcohol, tobacco, unhealthy food, and SSBs. Firstly, increases in price, restrictions on marketing, and reductions in availability are the key determinants of consumption and harm across all these policy areas [7]. Secondly, success in one policy area may lead to increased opportunities for harm in another if approached in isolation. For example, if alcohol sports sponsorship is banned, this could free up opportunities for sports sponsorship by fast food brands. Finally, data and evidence clearly demonstrate that multiple risks cluster, particularly among the poor, and this clustering results in a greater risk of disease and death [4–6]. As such, policy decisions that consider and respond to these risks together are likely to be more effective for preventing and reducing harm and narrowing health inequalities than policy decisions considering each risk in isolation.

Taxes on harmful commodities such as alcohol, tobacco, unhealthy foods, and SSBs are primarily levied to increase revenue but also play an important role in public health. Increasing the cost of the product relative to alternative spending choices and income reduces demand by decreasing affordability of the product which leads to lower levels of consumption and harm. Associated reductions in the cost of ill-health to society mean that well-designed taxes on harmful commodities can be a highly effective and cost-effective health intervention [7]. Standard economic theory predicts that a price increase will lead to a reduction in demand, which is typically measured using the PED (the percentage change in quantity demanded associated with a 1% change in price). Effectively designed taxes also provide a financial incentive for producers to reformulate their products to less harmful versions, for example, levying higher rates of tax on stronger alcohols might lead to reductions in product strength, and taxing excess fat in a product might lead to reductions in total fat content or replacement with alternative fats. Numerous reviews have examined the impact of price and taxes on consumption of alcohol, unhealthy food, SSBs, and smoking [18–28]. Given the behavioural, clinical, and policy synergies across these commodities, it is opportune to synthesise existing evidence to understand the similarities and differences in consumer price responses and what this might mean for bringing together tax policies. To the author’s knowledge, there has been one umbrella review which has attempted to do this, however included countries in Latin America only [29]. Therefore, the aim of this umbrella review was to identify and summarise evidence from systematic reviews that report the relationship between price and demand or price and disease/death for alcohol, tobacco, unhealthy food, and SSBs in any country. Since gambling is a taxable commodity and there have been recent efforts to conceptualise gambling using a public health approach [30, 31], we also considered gambling.

## Methods

The protocol for our umbrella review was pre-registered (PROSPERO CRD42023447429) [32] and our write up complies with the PRISMA 2020 reporting guidelines [33].

### Eligibility criteria

The PICO used in our umbrella review is given in Appendix 1. Eligible studies were peer-reviewed systematic reviews (with/without meta-analysis) published between 1 January 2000 and 17 July 2023 in any country. The start date was chosen to ensure elasticity estimates were applicable to the current policy context. Systematic reviews were defined as those which searched ≥ 2 electronic databases and reported search terms and eligibility criteria. Study samples had to reflect the general population and could include adults or children. Our protocol originally stated only adult samples were eligible (≥ 18 years), however several eligible reviews combined findings from adults and children, and it was not always possible to separate the results. As such, we updated our eligibility criteria to include all ages, and where possible, report findings for different populations separately. To be eligible, reviews had to report a quantitative association between the price/tax of alcohol, tobacco, unhealthy foods (those high in fat, salt, sugar), SSBs, or gambling, and demand or disease/death. Reviews including observational and experimental designs were eligible (modelling and qualitative designs were ineligible). Although price can be influenced by mechanisms other than taxes, such as distribution monopolies or vouchers, these non-tax regulations were ineligible to increase comparability across the included reviews. Reviews funded by the alcohol, tobacco, gambling, or food/drink industry were ineligible, as were reviews undertaken by known industry-funded actors. Reviews not funded by industry actors, however included authors listing industry conflicts of interest (CoI) were included, as were reviews which did not list their funding source or CoIs. Only English-language reviews were eligible.

### Information sources, search strategy and selection criteria

Ovid Medline, Ovid Embase, the Cochrane Database of Systematic Reviews, Epistemonikos, and EconLit (EBSCO) were searched from 1 Jan 2000 to 17 July 2023. An example search strategy is given in Appendix 2. IDEAS and EconPapers were searched from 1 Jan 2000 to 31 Dec 2022 because their results could not be downloaded, and we wanted to use reproducible searches when undertaking screening. Unpublished studies were not sought.

Records were screened using Rayyan [34]. Pilot title-and-abstract screening was undertaken by all researchers involved in screening on 100 records and indicated high levels of agreement (RB, SB, CH, MJ, MR, CS). Mean agreement was 92.7%, ranging from 90.0% to 97.0% across researcher pairs (Appendix 3). Thereafter, title-and-abstract screening was completed by a single researcher (RB), and a second checked a randomly selected 10% sample (SB, CH, MJ, CS). Although title-and-abstract screening was not undertaken in duplicate, if there was uncertainty about whether a record was eligible, we included it. There were high levels of agreement between screeners (ranging from 89.7% to 100% across researcher pairs), largely because it was easy to identify if a paper was a review or primary research study, and only systematic reviews were eligible. Full-text screening was completed in duplicate (RB, SB, CH, MJ, CS). Discrepancies were resolved by local discussion, consulting a third researcher if required.

### Data collection process and data items

One researcher (RB) extracted data using a standardised template and any uncertainties were discussed with the research team collectively (template given in Appendix 4). A second researcher (MJ, SB) fully checked the data extraction of 60% of included reviews, and additional spot checks were undertaken of the remaining 40%. Only data pertaining to our research question was extracted. For example, for a review including studies evaluating the impact of tobacco tax and a smoking ban, only information pertaining to tax was extracted. Similarly, if a review included observational and modelling studies, only data pertaining to observational studies was extracted. We requested missing data from authors, making a maximum of three attempts. We did not request country- or population-level information for individual studies in a review when high-level information was given, for example, in a review including high-income countries without specifying the exact countries.

### Study risk of bias assessment

In our protocol we proposed assessing risk of bias (RoB) using AMSTAR-2 [35], however, we changed our approach and used ROBIS [36]. This decision was guided by the fact that our definition of systematic reviews was taken from item 4 of the AMSTAR-2, and we excluded industry-funded research (item 16), rendering the tool less useful. We felt that ROBIS was preferable as we could consider the impact the methods had on the validity of findings, rather than assess whether a method had been correctly applied as is the case for AMSTAR-2 [37]. We note that AMSTAR-2 and ROBIS address similar, if not identical, methodological constructs and interrater reliability between the two tools is equivalent [38, 39]. RoB for all reviews was undertaken by a single researcher (CS) and a second researcher independently undertook a second appraisal of that review (RB, SB), and discrepancies were resolved by local discussion, consulting a third researcher if required. Completed RoB templates are available on request.

### Effect measures

The key outcome measure for this review was the PED or equivalent measure for the relationship between price/tax and demand/health outcomes. Where a meta-analysis had been carried out, the combined effect size was reported overall and by age, sex, and deprivation (if available). For systematic reviews which reported the PED (or equivalent) for each included study but did not undertake a meta-analysis, we extracted the range. For systematic reviews which only reported the direction of PED (or equivalent) for each included study and not the magnitude, we extracted the number of estimates reporting an inverse, positive, or no association. Although this approach captures the direction and not magnitude of effect, and overlooks important information about variability within estimates, it enabled us to include a broader range of reviews while ensuring uniformity in data extraction and interpretation. Additionally, this was a pragmatic alternative to extracting all missing information from primary studies which was not possible within our resource and time constraints.

### Synthesis methods

We undertook a narrative synthesis. Eligible reviews were grouped according to harmful commodity (alcohol, tobacco, unhealthy food, SSBs, gambling). Within these groups, we synthesised estimates for demand, and disease/death separately (measures of weight and body mass index (BMI) were included within the disease/death synthesis).

### Reporting bias assessments

Overall, 12 reviews had some missing information on the countries of the included studies, 10 had missing information on the age of included populations, and three did not provide a detailed definition of unhealthy food. This missing information is indicated by NR (not reported) in Table 1. Definitions of unhealthy food used within reviews were highly varied, but commonly included foods high in fat, salt, and/or sugar (HFSS). Where definitions were vague (e.g. “unhealthy food”), we included these reviews, despite a lack of clarity about exactly what foods were in scope.

**Table 1:** Summary table of included systematic reviews. The table is ordered alphabetically within commodities (alcohol, tobacco, unhealthy food, and sugar-sweetened beverages). Studies are organised alphabetically, within type of harmful commodity

Reference [review type]	N databases	N studies	Search period	Included languages	Included countries	Industry funding and CoI	Included outcomes		ROBIS rating
							**Demand**	**Disease / death**	
**Unhealthy food and soft drinks *n* = 31**									
**Sugar-sweetened beverages *n* = 13**									
Alagiyawanna 2015 [40] [SR]	6	9^a^	Inception-2013	ENG	HI = 9	None	O	O	U
Andreyeva 2022 [21] [SRMA]	8	35^b^	Inception-2020	Any	HI = 27, MI = 8	None	O		U
Backholer 2016 [41] [SR]	2	4^c^	Inception-2015	Any	HI = 4	None	O	O	L
Cabrera Escobar 2013 [19] [SRMA]	5	12	2000–2013	ENG	HI = 10, MI = 2	None	O	O	H
Hammaker 2022 [27] [SRMA]	12	17^d^	2000–2022	ENG	HI = 15, MI = 2	None	O	O	L
Itria 2021 [42] [SR]	5	16^e^	2009–2019	ENG ESP	HI = 12, MI = 4	None	O	O	H
Mackenbach 2022 [43] [SR]	3	1^f^	Inception-2021	ENG	MI = 1	None		O	H
Nakhimovsky 2016 [44] [SR]	6	7^g^	1990–2016	ENG	MI = 7	None	O	O	L
Nikniaz 2022 [45] [SR]	7	4^h^	2000–2021	ENG	HI = 4	None	O		L
Pérez-Ferrer 2019 [46] [SR]	3	3^i^	1999–2017	ENG ESP PRT	MI = 3	None	O		H
Redondo 2018 [47] [SR]	4	17	2011–2017	ENG ESP	HI = 15, MI = 2	Funding NR	O		H
Teng 2019 [20] [SRMA]	4	22	Inception-2018	Any	HI = 18, MI = 4	None	O		L
Von Philipsborn 2020 [48] [SR]	11	1^j^	Inception-2018	Any	HI = 1	CoI	O		L
**Unhealthy food *n* = 9**									
Andreyeva 2022 [49] [SR]	8	14^k^	Inception-2020	Any	HI = 8, MI = 6	None	O	O	H
Dodd 2020 [50] [SR]	4	6^l^	2000–2019	Any	HI = 3, MI = 3	None	O		H
Engler-Stringer 2014 [51] [SR]	9	1^m^	1995–2013	ENG	HI = 1	None	O		U
Holsten 2009 [52] [SR]	5	1^n^	Inception-2006	ENG	HI = 1	None		O	H
Lhachimi 2020 [53] [SR]	12	2	Inception-2009	Any	HI = 2	None	O		L
Mah 2019 [54] [SR]	3	18^o^	Inception-2018	ENG	HI = 18	None	O		H
Mizdrak 2015 [55] [SR]	5	6^p^	1980–2014	ENG	HI = 6	None	O		L
Pfinder 2020 [56] [SR]	12	1	Inception-2019	Any	HI = 1	None	O		L
Thow 2014 [57] [SR]	4	8^q^	2009–2012	ENG	HI = 8	Funding NR	O		H
**Unhealthy food and sugar-sweetened drinks combined *n* = 9**									
Afshin 2017 [18] [SRMA]	7	7^r^	1990–2014	NR	HI = 7	CoI	O		L
Green 2013 [22] [SRMA]	6	28^s^	Inception-2011	ENG	HI = 17, MI = 7, LI = 3, NR = 1	None	O		L
Mackenbach 2019 [58] [SR]	4	3^t^	Inception-2018	ENG NDL	HI = 2, MI = 1	None	O		L
Maniadakis 2013 [59] [SR]	6	41^u^	1990–2013	ENG	HI = 38, MI = 3	Unclear funding	O	O	H
Niebylski 2015 [60] [SR]	2	8^v^	2003–2013	ENG	HI = 7, NR = 1	CoI NR	O	O	U
Powell 2009 [61] [SR]	4	7^w^	1990–2008	ENG	HI = 7	Funding/CoI NR		O	H
Powell 2013 [62] [SR]	4	40^x^	2007–2012	ENG	HI = 40	CoI NR	O	O	H
Thow 2010 [63] [SR]	3	6^y^	2000–2009	ENG	HI = 5, MI = 1	Funding NR	O	O	H
Wright 2017 [64] [SR]	6	8^z^	1990–2015	ENG	NR = 8	None	O		H
**Tobacco = 9**									
Akter 2023 [65] [SR]	5	5^aa^	Inception-2021	ENG	HI = 4, MI = 1	None		O	L
Guindon 2015 [23] [SRMA]	4	22	Inception-2013	Any	MI = 15, LI/MI NR = 5, HI = 2	CoI NR	O		L
Hill 2014 [66] [SR]	12	7^bb^	2006–2010	ENG	HI = 6, NR = 1	None	O		H
Jawad 2018 [24] [SRMA]	4	8	Inception-2017	Any	HI = 8	None	O		H
Kjeld 2023 [67] [SR]	3	6	2011–2021	ENG	HI = 6	None	O		L
McKay 2015 [68] [SR]	7	1^cc^	Inception-2013	Any	MI = 1	None	O		L
Nazar 2021 [69] [SR]	5	28	Inception-2020	ENG	MI = 25, NR = 3	None	O		U
Thomas 2008 [70] [SR]	16	42^dd^	Inception-2006	Any	HI = 41, MI = 1	None	O		L
Wilson 2012 [71] [SR]	6	35^ee^	Inception-2009	Any	HI = 27, MI = 4, NR = 4	None	O		U
**Alcohol *n* = 9**									
Baldwin 2022 [72] [SR]	8	2^ff^	2010–2021	ENG	HI = 2	None		O	H
Elder 2010 [73] [SR]	7	78	Inception-2005	ENG	HI = 78	CoI NR	O	O	L
Kilian 2023 [25] [SRMA]	5	19^gg^	2000–2022	Any	HI = 15, MI = 3, NR = 1	CoI	O		L
Kõlves 2020 [74] [SR]	9	8^hh^	Inception-2019	ENG	HI = 6, MI = 2	None		O	L
Li 2015 [75] [SR]	5	2^ii^	1980–2013	ENG CHN	MI = 2	CoI NR	O	O	U
Scott 2017 [76] [SR]	7	1^jj^	Inception-2015	Any	HI = 1	None	O		L
Wagenaar 2009 [26] [SRMA]	9	112	Inception-2009	ENG	NR = 12	None	O		H
Wagenaar 2010 [28] [SRMA]	12	50	Inception-2009	ENG	HI = 47, NR = 3	CoI NR		O	L
Wilson 2014 [77] [SR]	11	2^kk^	1992–2013	ENG	HI = 2	None		O	H
**Multiple commodities *n* = 1**									
Miracolo 2021 [29] [SR]	5	14^ll^	2000–2018	ENG ESPN	MI = 10 HI = 3, NR = 1	Funding NR	O	O	H

Only data pertaining to our research question and eligibility criteria was extracted. As such, the number of studies synthesised in our review might not match the number of studies listed in the published review. The number of studies listed in published reviews was as follows: ^a^18, ^b^86, ^c^11, ^d^51, ^e^21, ^f^23, ^g^9, ^h^59, ^I^84, ^j^58, ^k^54, ^l^18, ^m^26, ^n^7, ^o^86, ^p^8, ^q^43, ^r^26, ^s^136, ^t^43, ^u^55, ^v^78, ^w^9, ^x^47, ^y^24, ^z^102, ^aa^144, ^bb^84, ^cc^80, ^dd^84, ^ee^84, ^ff^31, ^gg^39, ^hh^19, ^ii^21, ^jj^48, ^kk^23, ^ll^34

*CHN* Chinese, *CoI* conflict of interest, *ENG* English, *ESPN* Spanish, *H* high risk of bias, *L* low risk of bias, *PRT* Portuguese, *NDL* Danish, *NR* not reported, *ROBIS* risk of bias in systematic reviews, *SR* systematic review, *SRMA* systematic review and meta-analysis, *U* unclear risk of bias

We requested missing information from four authors regarding the number and names of databases used [70], search strategies [70, 76, 78], and language-based eligibility criteria [18] and received requested information from two authors [70, 76]. We did not receive a search strategy for one review [78] and, because our eligibility criteria for systematic reviews requires reporting search terms, it was then excluded.

To explore the potential impact of industry CoIs or missing CoI or funding information, we compared reviews without industry funding/CoIs to reviews with industry CoIs or missing information.

### Interpretation of results

Through informal verbal discussions within the research team, interpretation of the results was guided by the four components in GRADE-CERQual (methodological limitations, coherence, adequacy, and relevance) [79]. This helped us to take a structured approach to synthesising the data across the different commodities and frame the discussion. Methodological limitations were assessed using the ROBIS scores, focusing on sources of bias and what that meant for the findings of the included reviews. When assessing coherence of findings, we did this between reviews within a single commodity of interest (such as alcohol), as well as across reviews of different commodities (alcohol, tobacco, unhealthy food, and SSBs). This helped us identify whether the data from contributing studies provided a plausible explanation for the relationship between price and demand not only for specific products, but as a general mechanism of action. Our discussions about the adequacy of the data revealed the limitations of using review-level evidence which lacked a richness, however had the trade-off that a larger quantity of data was included (noting overlap in the primary studies included across the reviews). Relevance was considered while undertaking screening, since our eligibility criteria were restricted to typical populations and settings so their findings could be more easily generalised to the general population.

## Results

### Study selection

**Fig. 1:**
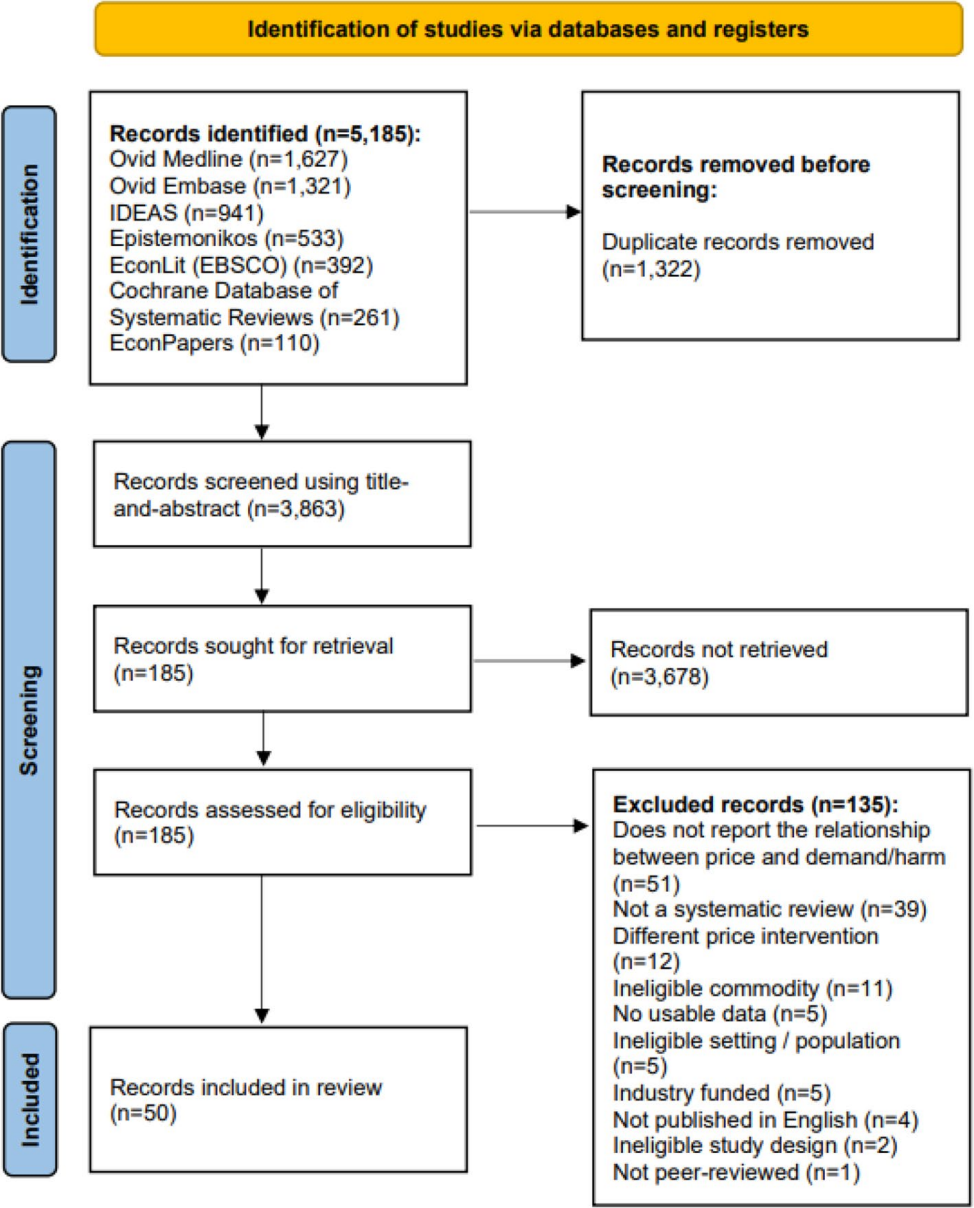
PRISMA flow diagram of screening and selection process

The search returned 5,185 records, of which 3,863 were screened for eligibility using title-and-abstract (Fig. 1). A total of 185 were screened using full-texts, and 50 were eligible and included in this umbrella review. Examples of excluded records and reasons for their exclusion are given in Appendix 5.

### Study characteristics

A summary of the commodities of interest, included outcomes, and methodological aspects covered across the included systematic reviews is given in Table 1. Of the 50 included reviews, 31 reported on unhealthy food or SSBs [18–22, 27, 40–58, 60–64, 80], nine reported on tobacco [23, 24, 65–71], nine on alcohol [25, 26, 28, 72–77], and one on multiple outcomes (alcohol, tobacco, and SSBs) [29]. Included reviews on unhealthy food and/or SSBs were generally published more recently than reviews on alcohol and tobacco: almost half of the alcohol and tobacco reviews were published before 2014, whereas almost half of the unhealthy food and/or SSBs reviews were published after 2017 (Appendix 6). No reviews were identified for gambling. Most reviews reported on the price-demand response (*n* = 42) with fewer reporting on the price-disease/death response (*n* = 22). Over 80% of reviews included mostly high-income countries, and there was a mix of adult/child samples (Table 1).

### Risk of bias in studies

**Fig. 2:**
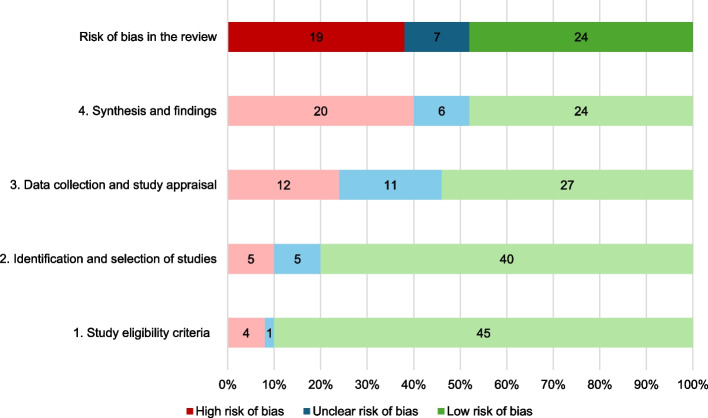
Risk of bias (RoB) across the included systematic reviews using ROBIS

Of the 50 systematic reviews included in this umbrella review, 24 were rated as having a low RoB, 18 as having a high RoB, and eight as having an unclear RoB (Fig. 2, Appendix 7). Common sources of bias included poor handling or reporting of variability and robustness of findings, and a lack of detail or ambiguity about screening, data collection, and quality appraisal procedures. RoB scores for each review and by commodity (alcohol, tobacco, and unhealthy food/SSBs) are available in Appendix 7 which includes a description of the main sources of bias for each review and our approach to scoring.

### Results of individual studies

**Fig. 3:**
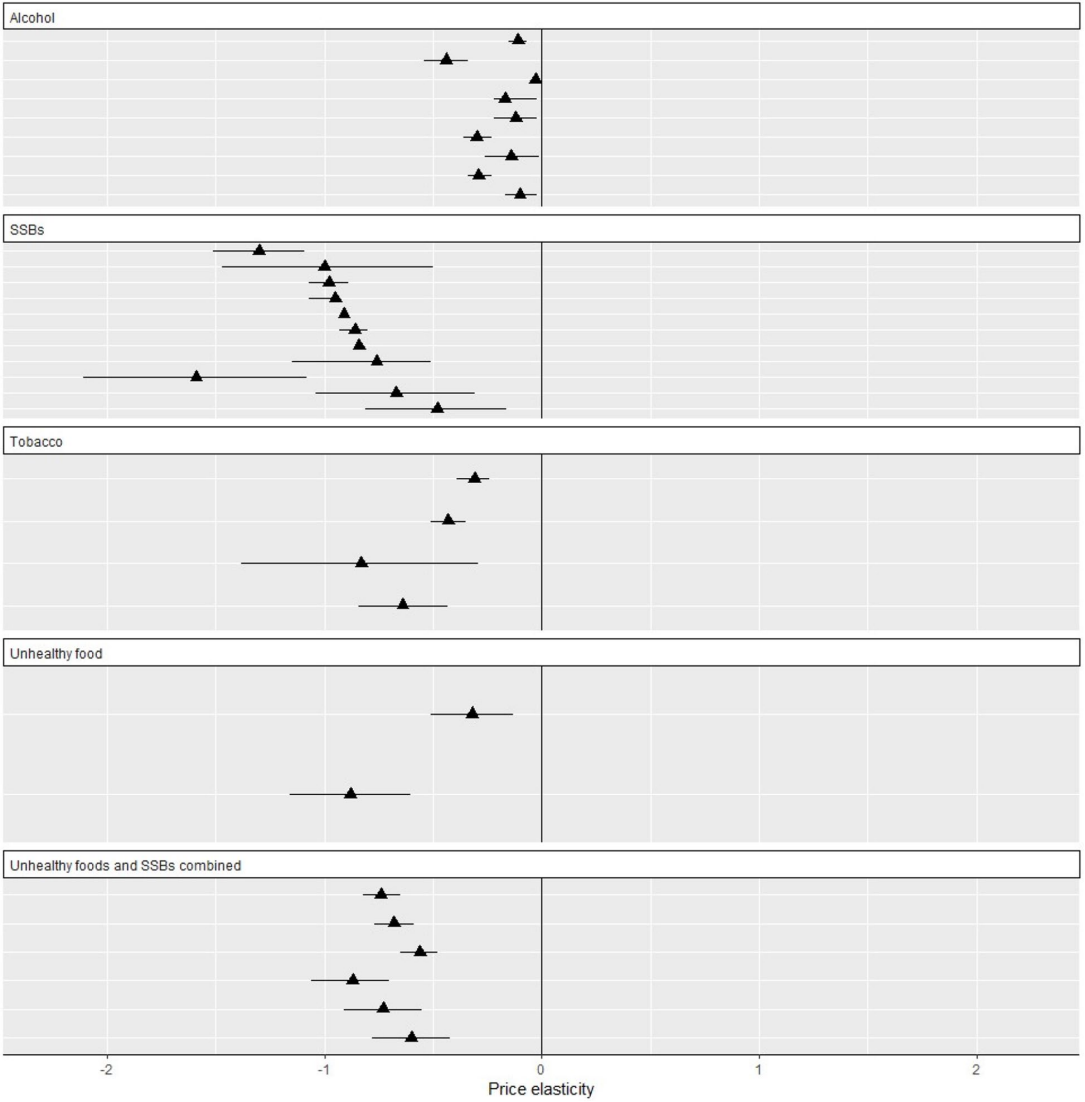
Scatterplot showing the association between price and demand for alcohol, tobacco, unhealthy food, and sugar-sweetened beverages. Each data point represents an estimate from included meta-analyses. There will be an overlap in studies contributing to these estimates

An overview of included systematic reviews which undertook meta-analyses for the relationship between price and demand are given in Table 2, with a visual depiction in Fig. 3. An overview of included systematic reviews which undertook a narrative synthesis are given in Appendix 8 (for demand), Appendix 9 (for disease/death) and Appendix 10 (for impacts on deprived groups).

**Table 2:** Overview of findings reported in included systematic reviews which undertook meta-analyses. Tables are ordered within commodities from low to high risk of bias

Study author (ref); search period	Study design n; population n	Intervention	Subgroup (if applicable)	Pooled PED (95% CI)	ROBIS score
**Systematic reviews with meta-analyses for SSBs *n* = 4**					
Afshin 2017 [18] 1990 – 2014	Observational = 7 All ages = 3, children = 3, adults = 1	SSB and other unhealthy drinks (NS) price/tax	SSBs	-0.67 (-1.04, -0.31)	Low RoB
			Other unhealthy drinks	-0.48 (-0.81, -0.16)	
Andreyeva 2022 [21] Inception – 2020	Observational = 35 NR, all ages were eligible	SSB price/tax	SSB sales	-1.59 (-2.11, -1.08)	Low RoB
			SSB consumption	-3.78 (-8.86, 1.30)	
Teng 2019 [20] Inception – 2018	Observational = 22 All ages = 22	SSB tax	Overall	-1.00 (-0.50, -1.47)	Low RoB
			USA states (excluding Berkely)	-0.98 (-0.89, -1.07)	
			Berkeley	-0.95 (-0.93, -0.98)	
			Mexico	-0.91 (-0.90, -0.92)	
			Catalonia	-0.86 (-0.80, -0.93)	
			France	-0.84 (-0.84, -0.85)	
			Chile	-0.76 (-0.51, -1.15)	
Cabrera Escobar 2013 [19] 2000 – 2013	Observational = 12 All ages = 8, adults = 3, children = 1	SSB price/tax	-	-1.30 (-1.09, -1.51)	High RoB
**Systematic reviews with meta-analyses for unhealthy foods and SSBs combined *n* = 2**					
Afshin 2017 [18] 1990 – 2014	Observational = 7 All ages = 3, children = 3, adults = 1	Unhealthy food (NS) and SSB price/tax	All unhealthy food and SSBs	-0.60 (-0.78, -0.42)	Low RoB
Green 2013 [22] Inception – 2011	Observational = 28 NR, all ages were eligible	Sweets, confectionary and SSB price/tax	Low-income countries	-0.74 (-0.82, -0.65)	Low RoB
			Middle-income countries	-0.68 (-0.77, -0.59)	
			High-income countries	-0.56 (-0.65, -0.48)	
			Lowest-income households	-0.87 (-1.06, -0.70)	
			Highest-income households	-0.73 (-0.91, -0.55)	
**Systematic reviews with meta-analyses for unhealthy foods *n* = 1**					
Afshin 2017 [18] 1990 – 2014	Observational = 7 All ages = 3, children = 3, adults = 1	Unhealthy food (NS)	Fast foods	-0.32 (-0.51, -0.13)	Low RoB
			Other unhealthy food	-0.88 (-1.16, -0.60)	
**Tobacco systematic reviews with meta-analyses *n* = 2**					
Guindon 2015 [23] Inception – 2013	Observational = 22 All ages = 22	Tobacco price/tax	Short-run	-0.31 (-0.39, -0.24)	Low RoB
			Long-run	-0.43 (-0.51, -0.35)	
Jawad 2018 [24] Inception – 2017	Observational = 8 NR, all ages were eligible	Cigars and hand-rolled tobacco price/tax	Cigars	-0.83 (-1.38, -0.29)	High RoB
			Hand-rolled tobacco	-0.64 (-0.84, -0.43)	
**Alcohol systematic reviews with meta-analyses *n* = 2**					
Kilian 2023 [25] 2000 – 2022	Observational = 19 All ages = 18, adults = 1	Alcohol price/tax	-	-0.11 (-0.15, -0.07)^a^	Low RoB
Wagenaar 2009 [26] Inception – 2009	Observational = 112 NR, all ages were eligible	Alcohol price/tax	All alcohol: aggregate studies	-0.44 (-0.54, -0.34)	High RoB
			All alcohol: individual studies	-0.03 (-0.05, -0.02)	
			Beer: aggregate studies	-0.17 (-0.22, -0.12)	
			Beer: individual studies	-0.12 (-0.22, -0.02)	
			Wine: aggregate studies	-0.30 (-0.36, -0.23)	
			Wine: individual studies	-0.14 (-0.26, -0.01)	
			Spirits: aggregate studies	-0.29 (-0.34, -0.23)	
			Spirits: individual studies	-0.10 (-0.17, -0.02)	

Only data pertaining to our research question and eligibility criteria was extracted. For example, if a review undertook a meta-analysis of studies evaluating both the impact of a tobacco tax and a smoking ban, only information pertaining to tax was extracted. As such, the number of studies listed in this table might not match the numbers listed in the published review. Additional information about each review can be seen in Table 1. We identified an additional meta-analysis which was not included in this table because it did not report PED, however the results were consistent and showed that an SSB tax was associated with a decrease in demand [27]

*CI* confidence interval, *NR* not reported, *NS* not specified, *PED* price elasticity of demand, *RoB* risk of bias, *ROBIS* risk of bias in systematic reviews, *SSB* sugar-sweetened beverage

^a^The review reports change for a 100% increase in price which we have converted to a 1% change for comparability across reviews

## Results of syntheses

### The relationship between price and demand

The included systematic reviews find that increases in the price of alcohol, tobacco, unhealthy food, or SSBs are associated with decreases in demand, notwithstanding variation in the size of effect across commodities or populations (Fig. 3, Appendix 8). An inverse association between price and demand was consistently seen across all outcomes. Looking across the included meta-analyses, a 10% increase in product price was associated with a median reduction in demand of 9.1% for SSBs [18–21], 6.0% for unhealthy food [18], 5.4% for tobacco [23, 24], and 1.4% for alcohol [25, 26]. It should be noted that there will be overlap in the studies contributing to these estimates which we did not quantify. Where reviews reported the differential impact of price on different groups, the evidence generally suggests that more deprived groups are more price responsive than less deprived groups (Appendix 10).

### The relationship between price and disease or death

Compared to the body of evidence reporting the relationship between price and demand, the body reporting the relationship between price and disease/death was smaller (Appendix 9). Across the alcohol reviews, inverse relationships were most consistently seen for outcomes which are wholly caused by alcohol (those with an alcohol attributable fraction of 1), such as alcohol-related liver disease or alcohol dependence [28, 73–75]. The direct reporting of these outcomes enables accurate tracking of trends, whereas trends in partially attributable outcomes may be obscured by other factors. One systematic review including a small number of estimates suggested an increase in the price of tobacco was associated with a decrease in cases of lung cancer, respiratory disease, and cardiovascular disease, however there was large between-studies variability [65]. An emerging body of evidence supports an inverse association between the price of SSBs and the prevalence of dental caries [27, 43].

### Reporting biases

Table 1 shows funding and CoI information listed in reviews. When we compared the results of reviews which did not included a funding or CoI statement (*n* = 11), had industry CoIs (*n* = 3), or had unclear funding (*n* = 1), to the results of reviews with no industry funding or CoIs (*n* = 35), we found no discernible differences in the magnitude or direction of associations.

## Discussion

We undertook a comprehensive umbrella review of systematic reviews to identify the relationship between the price of alcohol, tobacco, unhealthy food, SSBs, or gambling, and demand or disease/death. Despite alcohol and tobacco being taxable commodities for centuries [81, 82], we identified fewer reviews in these areas compared to reviews on unhealthy food and SSBs which have only recently been viewed as taxable commodities [83, 84]. We did not identify any reviews for gambling. Published evidence on the effectiveness of tax as a measure to prevent and reduce gambling-related harms is limited, however its potential use as a public health tool has been debated in recent scientific papers and the results of an e-Delphi consensus study highlight them as a possibly effective public health policy [31]. Goyder and colleagues argued that gambling taxation might work in a similar way to alcohol, tobacco, and SSB tax [85], and Sulkunen and colleagues suggest that increases in gambling tax might make it less profitable to providers, which may reduce their interest in expanding [86]. Others have suggested that gambling taxes might have a harmful impact by increasing the losses of gamblers, and it is excessive losses which mediate gambling-related harm, not the gambling itself [87]. UK estimates of the PED for gambling range from -1.5 for remote gaming to -0.5 for betting pools [88].

Our review clearly demonstrates that, at the most basic level, price interacts with income to affect demand, such that price increases of alcohol, tobacco, unhealthy food, or SSBs are associated with reductions in demand. Such consistency in findings clearly highlights the fundamental role of tax as a public health policy. Our review suggests that a 10% increase in product price is associated with reductions in demand in the order of 9.% for SSBs, 6% for unhealthy food, 5% for tobacco, and 1% for alcohol. This inverse relationship was most consistently seen for alcohol, and least consistently for SSBs. The relationship between price and demand was seen among adult and child populations, with some review-level evidence to suggest that children and young people were more price responsive in the case of tobacco [67, 71], as might also be the case for SSBs [20], although most reviews did not explicitly make this comparison. Although most reviews included research from high-income countries, where studied, the inverse price-demand relationship was seen across low-, middle-, and high-income countries. There was a lack of research on heavy consumers. Although the reviews present a consistent overall picture of the impact of price/tax on demand, there was large variation in estimates across the individual studies which may relate to methodological differences across studies. Elasticity estimates also vary over time, with some suggestion that products have become less price elastic, which probably reflects increasing affluence as products become more affordable [89].

Reviews on tobacco, unhealthy food, and SSBs generally provided evidence that lower income, education, or socioeconomic status groups were more price responsive compared to their less deprived counterparts for example [27, 47, 55, 66], but no alcohol reviews included this evidence. Nonetheless, data from primary research studies suggests that more deprived drinkers are more price responsive [90, 91]. A common argument against the imposition of taxes is that they may have a proportionally greater financial impact on people with lower incomes relative to those with higher incomes. However, lower income groups as a whole reap greater health benefits [92, 93]. To the extent that lower income individuals are more price sensitive, they will be more likely to cut back on the intake of taxed commodities, often starting from a higher level of consumption, and thus experience greater health gains.

Raising taxes not only has direct public health benefits but can also generate considerable revenue for governments. If price increases do not respond proportionately to tax increases, (i.e. if the PED < 1), government revenue will increase when taxes increase because the decrease in consumption is more than offset by the extra tax paid by those who continue to purchase the taxed product. Our review suggests this is the case for alcohol, tobacco, and unhealthy food, however some of the PED estimates for SSBs exceed one, suggesting government revenue might reduce because of the greater demand response. Nonetheless, SSB taxes are not widely implemented so any revenue generated by their introduction is revenue that would not have otherwise existed. Increased revenue associated with tax increases may go some way in compensating for the societal and human costs associated with alcohol, smoking, and excess weight.

Risky alcohol use, smoking, and excess weight, commonly co-occur and cluster in the most deprived [4, 5]. Multiple risks also result in large risks of disease or death which are synergistic in the case of alcohol and excess weight for liver disease and alcohol and smoking for oral cancers [6]. From these perspectives (behavioural and clinical), there is clear rationale for joining up policy approaches to prevent and reduce harm. The results of our review further highlight the potential of joining up policy approaches, specifically taxation and price increases. While taxation shares the same mechanism of action for alcohol, tobacco, unhealthy food, and SSBs, it should be noted that the end-goal of policy implementation might differ. An appropriate goal for smoking is usually complete abstinence, whereas for alcohol, the aim is to decrease the number of people drinking at levels which increase their risk of health harm (noting abstinence might be the goal for people with alcohol dependence). Although the WHO have recently recognised that there is no safe level of alcohol consumption [94], in practice, most countries have developed low-risk drinking guidelines at levels above zero which are typically set at what is considered to be an acceptable level of risk [95–97]. Diet however is multifaceted and may comprise aspects such as consuming fruit and vegetables, whole-grain high-fibre foods, and limiting sugar, fat, and salt. While this umbrella review has demonstrated the effectiveness of tax as a public health measure for alcohol, tobacco, unhealthy food, and SSBs in isolation, it has not been able to explore the impact of simultaneous tax increases across these commodities, namely because the evidence does not exist. Future research should aim to understand the potential public health impact of a holistic approach to tax policy spanning these commodities.

### Strengths and limitations

We identified reviews according to a pre-published protocol, focusing on the harmful commodities responsible for the largest burden of overall ill-health. As with all types of information retrieval there is a risk of overlooking relevant literature. Although title-and-abstract screening was not done in duplicate, levels of agreement between raters was high, thereby reducing this risk. Additionally, we consulted with topic experts at the Office for Health Improvement and Disparities to ensure there were no major oversights. Several reviews were based on a similar pool of primary studies and are therefore not independent. We did not attempt to identify levels of overlap between reviews however duplication is likely to be highest in reviews of SSBs given the larger numbers of reviews with similar aims and eligibility criteria.

The amount of detail included in reviews was highly variable limiting our ability to meaningfully compare effect sizes across reviews. To overcome this, where exact estimates were not reported, we extracted the number of estimates reporting an inverse, positive, or no association. Although this enabled us to include a wider number of relevant reviews and develop an understanding of the consistency of directional effects, this limited our ability to understand the magnitude of effect or capture explanations for mixed or null effects. Nonetheless, we gained insight into the magnitude of effect from meta-analyses and the range in PED in systematic reviews which did report effect sizes.

The impact of price increases can be mitigated by consumers substituting products which have experienced a price increase for others which have not. Exploring this ‘cross-price elasticity’ was outside the scope of this review, however we note evidence that UK consumers tend to treat off-trade wine and cider as substitutes, meaning consumers are happy to switch from wine to cider if the price of wine increases [98], and higher prices for SSBs are associated with an increased demand for fruit juice [19]. Increases in the price of cigarettes has been shown to lead to substantial increases in per-capita sales of nicotine replacement products [99], and with the advent of electronic-cigarettes, emerging research suggests they are partially substitutable for combustible cigarettes [100]. Such commodity shifting should be kept in mind when implementing any taxation policy. We also note that our review only demonstrates the effectiveness of price policy for alcohol, tobacco, unhealthy food, and SSBs in isolation, and does not consider evidence of the impact of a combined taxation approach.

We did not assess the risk of bias in individual studies included in each review, and only undertook these appraisals at the review level. We note that 19 of the 50 reviews (38%) had a high RoB and a further eight had an unclear RoB (16%), which should be borne in mind when considering the validity of the results. It is possible that researchers had flaws in their methodological approach, however, standardised reporting guidelines such as PRISMA were only published in 2009 (with an update in 2020) and their use has picked up greatly in more recent years [33, 101]. Some of the biases in reviews arose due to reporting issues where information was either not included, or unclear, so it is likely that some of these low scores relate to reporting rather than actual methodological quality. This would especially be the case for older reviews that were published before PRISMA was widely adopted, which in our sample were more likely to be reviews on tobacco or alcohol.

## Conclusion

While risky alcohol consumption, smoking, and excess weight all represent a substantial public health burden in and of themselves, there are clear behavioural, clinical, and policy synergies across these risks. The evidence supports tax/price increases as effective policies for reducing demand, which suggests there might be merit in a joined-up approach to tax, with the treasury representing an arbiter of public health and the National Health Service (NHS) costs.

### Supplementary Information

**Supplementary Material 1.**

## Data Availability

Data sharing is not applicable to this article as no datasets were generated or analysed during the current study. Full data extraction of studies included in this umbrella review are available on request by contacting robyn.burton@stirling.ac.uk.

## Abbreviations

BMI: Body mass index
CI: Confidence interval
COI: Conflicts of interest
DALYs: Disability adjusted life years
HFSS: High in fat, salt and/or sugar
PED: Price elasticity of demand
RoB: Risk of bias
SSB: Sugar sweetened-beverage
