# Supplementary material for: The relationship between the price and demand of alcohol, tobacco, unhealthy food, sugar-sweetened beverages, and gambling: an umbrella review of systematic reviews
Source: BMC Public Health. 2024 May 10;24:1286. doi: 10.1186/s12889-024-18599-3 (PMC11088175; doi:10.1186/s12889-024-18599-3)
Supplement: Supplementary file 1 — Supplementary Material 1. [file 12889_2024_18599_MOESM1_ESM.docx]

**Appendix 1: Description of participants, intervention, comparison, outcomes, study designs, time frame, and language.**

| **Criteria** | **Included** | **Excluded** |
| --- | --- | --- |
| **Population** | Adults or children | Atypical populations such as prisoners, military personnel, pregnant women etc |
| **Setting** | Low, middle, or high-income countries | Atypical settings such as prisons, airports etc |
| **Intervention** | Tax or retail price of unhealthy foods/soft drinks (those high in sugar, fat, or salt), alcohol, smokable tobacco, or gambling | Import/export taxes  In-kind provision (e.g., free school meal handouts) or subsidised products  Vouchers or cash transfers/cashbacks/food stamps  Fiscal policies that are not taxes, such as price floors/ceilings |
| **Comparison** | Change in tax / price | Studies without a change in tax / price |
| **Outcome(s)** | Demand (sales / consumption / prevalence)  Weight  Morbidity / mortality (prevalence, incidence, life expectancy) | Outcomes unrelated to demand, weight, or morbidity / mortality |
| **Study designs** | Systematic reviews including at least one experimental or quasi-experimental evaluation. Reviews of interventions and associations were included  Systematic reviews were defined as those with report their inclusion / exclusion criteria, used 2+ electronic databases, and reported their search terms | Systematic reviews including only qualitative designs or modelling studies |
| **Time frame** | No restriction | - |
| **Language** | English | Not English |

Only data pertaining to our research question and eligible was extracted. For example, if a review undertook a meta-analysis of studies evaluating both the impact of a tobacco tax and a smoking ban, only information pertaining to tax was extracted. Similarly, if a review included both modelling and observational studies, only the data pertaining to observational studies were included.

**Appendix 2: An example of our literature search strategy (all search strategies are available on request).**

**Ovid Embase**

1 elasticit*.tw,kf.

2 (price or prices or pricing).tw,kf.

3 (cheap or cheaper or cheapest or expensive or spend*).tw,kf.

4 (own-pric* or cross-pric*).tw,kf.

5 (subsidy or subsidies).tw,kf.

6 discount*.tw,kf.

7 (tax or taxes or taxation).tw,kf.

8 (levy or levies or duty or duties).tw,kf.

9 ("cost" or "costs").ti.

10 purchas*.tw,kf.

11 (sale or sales).tw,kf.

12 expenditure.tw,kf.

13 affordab*.tw,kf.

14 demand.tw,kf.

15 fiscal polic*.tw,kf.

16 exp tax/

17 "cost"/

18 fiscal policy/

19 consumer attitude/

20 commercial phenomena/

21 exp price/

22 1 or 2 or 3 or 4 or 5 or 6 or 7 or 8 or 9 or 10 or 11 or 12 or 13 or 14 or 15 or 16 or 17 or 18 or 19 or 20 or 21

23 diet*.tw,kf.

24 high fat.tw,kf.

25 food*.tw,kf.

26 drink*.tw,kf.

27 beverage*.tw,kf.

28 soda.tw,kf.

29 salt.tw,kf.

30 sugar*.tw,kf.

31 alcohol.tw,kf.

32 (beer* or wine*).tw,kf.

33 spirit*.tw,kf.

34 tobacco.tw,kf.

35 smok*.tw,kf.

36 cigar*.tw,kf.

37 gambl*.tw,kf.

38 Food/

39 Carbonated Beverage/

40 exp alcoholic beverage/

41 Gambling/

42 lipid diet/

43 drinking behavior/

44 Tobacco/

45 smoking/ or cigar smoking/ or cigarette smoking/

46 salt intake/

47 Sugar-Sweetened Beverage/

48 sugar intake/

49 Fast Food/

50 or/23-49

51 22 and 50

52 limit 51 to yr="2000 - 2023"

53 limit 52 to "reviews (maximizes specificity)"

54 limit 53 to conference abstracts

55 53 not 54

**Appendix 3: Inter-rater agreement.**

**Inter-rater agreement for pilot screening between researcher pairs.**

| **Researcher** | **RB** | **SB** | **CH** | **MJ** | **MR** | **CS** |
| --- | --- | --- | --- | --- | --- | --- |
| **RB** |  | 92% | 94% | 91% | 90% | 97% |
| **SB** |  |  | 94% | 91% | 96% | 91% |
| **CH** |  |  |  | 91% | 94% | 91% |
| **MJ** |  |  |  |  | 93% | 92% |
| **MR** |  |  |  |  |  | 93% |
| **CS** |  |  |  |  |  |  |
| Mean agreement was 92.7%, ranging from 90.0% to 97.0% across researcher pairs | | | | | | |

**Agreement for title-and-abstract screening.**

| **Title-and-abstract section** | **Number of records** | **First researcher** | **Second researcher** | **% agreement** |
| --- | --- | --- | --- | --- |
| **1** | 282 | RB | MR | 93.1% |
| **2** | 282 | CS | CH | 100.0% |
| **3** | 281 | SB | CS | 93.1% |
| **4** | 281 | RB | SB | 93.1% |
| **5** | 281 | MJ | SB | 93.1% |
| **6** | 281 | RB | CH | 89.7% |
| **7** | 281 | RB | MR | 100.0% |
| **8** | 281 | RB | CS | 96.6% |
| **9** | 281 | MR | CS | 93.1% |
| **10** | 281 | RB | MR | 96.6% |
| **11** | 110 | RB | CS | 90.9% |
| **12** | 941 | RB | CS | 93.7% |
| Mean agreement was 94.4%, ranging from 89.7% to 100.0% across researcher pairs | | | | |

**Appendix 4: Data extraction template.**

| **Data item** | **Brief description of data item** |
| --- | --- |
| **Reference, type of review** | Include the reference for the review and state whether it is a systematic review or systematic review and meta-analysis |
| **Eligible dates** | Include the search dates of the electronic databases |
| **Included study designs (study n)** | Identify the number of studies included in the review and the number that used observational or experimental designs |
| **Country (study n)** | Identify the number of studies in the review from each country |
| **Population (study n)** | Identify the number of studies in the review which used child, adult, or all ages samples |
| **Intervention [retail price/tax of alcohol, tobacco, unhealthy food, sugar-sweetened drinks]** | Identify the intervention of interest (retail price/tax) and commodity (alcohol, tobacco, unhealthy food, sugar-sweetened beverages) |
| **Outcome(s)** | Demand, disease, or death |
| **Main results** | If a meta-analysis, extract the pooled PED and 95% CIs overall and by age, sex, or deprivation status (if available). If a systematic review, extract the range in PED (if PED is reported for all included studies). If PED is not reported for all included studies, extract the number of estimates that report an inverse, positive, no, or mixed association |
| **Conflicts of interest ^a^** | Identify whether there are industry related conflicts of interest |
| ^a^ Reviews funded by the alcohol, tobacco, food, or soft drinks industry or known industry-funded actors were not eligible, however we did include reviews which did not report their funding or reported industry conflicts of interest. We included reviews which synthesised individual industry-funded studies or authors with industry conflicts of interests, if the reviews themselves complied with the above. | |

**Appendix 5: Examples of excluded records alongside reasons for their exclusion and exclusion hierarchy.**

Records were excluded according to a hierarchy, selecting the reason highest up the list to complete the PRISMA flow diagram. In practice, a review may be ineligible for multiple reasons. A full list of excluded papers and their reasons is available on request.

**Screening hierarchy of exclusions reasons and example references**

| **(Order) Reason for exclusion** | **Example reference** |
| --- | --- |
| 1. Not published in English | Huybrechts I, De Bourdeaudhuij I, Buck C, De Henauw S. Environmental factors. Opportunities and barriers for physical activity and healthy eating among children and adolescents. Bundesgesundheitsblatt, Gesundheitsforschung, Gesundheitsschutz. 2010 Jul 1;53(7):716-24. |
| 2. Not a systematic review ^a^ | Galizzi MM. Label, nudge or tax? A review of health policies for risky behaviours. Journal of public health research. 2012 Mar;1(1):jphr-2012. |
| 3. Does not report the relationship between price and demand/harm | Munt AE, Partridge SR, Allman‐Farinelli M. The barriers and enablers of healthy eating among young adults: A missing piece of the obesity puzzle: A scoping review. Obesity reviews. 2017 Jan;18(1):1-7. |
| 4. Ineligible commodity ^b^ | Mogendi JB, De Steur H, Gellynck X, Makokha A. Consumer evaluation of food with nutritional benefits: a systematic review and narrative synthesis. International journal of food sciences and nutrition. 2016 May 18;67(4):355-71. |
| 5. Industry funded ^c^ | Nelson JP. Meta-analysis of alcohol price and income elasticities–with corrections for publication bias. Health economics review. 2013 Dec;3:1-0. |
| 6. No usable data ^d^ | Mozaffarian D, Afshin A, Benowitz NL, Bittner V, Daniels SR, Franch HA, Jacobs Jr DR, Kraus WE, Kris-Etherton PM, Krummel DA, Popkin BM. Population approaches to improve diet, physical activity, and smoking habits: a scientific statement from the American Heart Association. Circulation. 2012 Sep 18;126(12):1514-63. |
| 7. Different price intervention ^e^ | Boniface S, Scannell JW, Marlow S. Evidence for the effectiveness of minimum pricing of alcohol: a systematic review and assessment using the Bradford Hill criteria for causality. BMJ open. 2017 May 1;7(5):e013497. |
| 8. Ineligible setting / population ^f^ | Driessen CE, Cameron AJ, Thornton LE, Lai SK, Barnett LM. Effect of changes to the school food environment on eating behaviours and/or body weight in children: a systematic review. Obesity reviews. 2014 Dec;15(12):968-82. |
| 9. Not peer-reviewed | DeCicca P, Kenkel D, Lovenheim MF. The economics of tobacco regulation: a comprehensive review. Journal of economic literature. 2022 Sep 1;60(3):883-970. |
| 10. Ineligible study design ^h^ | Eyles H, Ni Mhurchu C, Nghiem N, Blakely T. Food pricing strategies, population diets, and non-communicable disease: a systematic review of simulation studies. PLoS medicine. 2012 Dec 11;9(12):e1001353. |
| ^a^ To be considered systematic, a review had to search 2+ electronic databases, report the search terms used, and report eligibility (inclusion/exclusion criteria)  ^b^ Only alcohol, tobacco, unhealthy food, sugar-sweetened beverages, or gambling were eligible  ^c^ Research that was directly funded by the alcohol, tobacco, food/drink, or gambling industry  ^d^ Insufficient information was reported which prevented us from extracting usable data  ^e^ Only changes in retail price/tax were eligible. Non-tax interventions were outside of scope, e.g. vouchers, subsidies, minimum unit price etc.  ^f^ Settings and samples had to reflect those alcohol, tobacco, unhealthy food/drink, and gambling is typically purchased and generalisable groups, e.g. airports, prisons, pregnant women, etc were ineligible  ^g^ Non-generalisable samples and settings were excluded, e.g. prisoners, pregnant women, people with complex mental health problems etc.  ^h^ Reviews had to include at least one study which used either an experimental or observational design | |

**Appendix 6: Included systematic reviews by publication year and commodity.**

The alcohol and tobacco literatures were generally older than the literature published on unhealthy food and/or sugar-sweetened beverages. Almost half of the included alcohol and tobacco reviews were published before 2014, whereas almost half of the included reviews on unhealthy food and/or sugar-sweetened beverages were published after 2017.

**The cumulative number of included reviews by publication year and commodity.**

MA: meta-analysis published

**Appendix 7: Risk of bias scores based on the Risk of Bias in Systematic Reviews (ROBIS) tool.**

ROBIS has four domains that are used to identify concerns with a review including study eligibility, identification and selection of studies, data collection and study appraisal, and synthesis and findings. In each domain, there are five to six signalling questions which are used to assess the domain as low, high, or unclear risk of bias (RoB). Answers of “Yes” or “Probably Yes” signal low RoB, “No” or “Probably No” signal high RoB, and “No Information” signals unclear RoB. The review’s overall RoB score considered each domain’s score as well as interpretation of the review findings and limitations. We developed a scoring rubric to ensure a consistent approach was taken to scoring domains as follows:

- If all questions scored “Yes” or “Probably Yes”, this domain was scored as low RoB
- If one question scored “No information”, “No”, or “Probably No”, this domain was scored as low RoB
- If two questions scored “No” or “Probably No”, this domain was scored as high RoB
- If two questions or more questions scored “No information”, this domain was scored as unclear RoB
- If one question scored “No” or “Probably No” and one question scored “No information”, the domain score was decided on a case-by-case basis following discussions with the review team and while considering the sources of bias

Our approach to scoring the overall RoB was as follows:

- If all four domains scored low RoB, the overall score was low RoB
- If one domain scored unclear or high RoB, or all domains had a mix of unclear and high RoB scores, these reviews were assessed on a case-by-case basis following discussions with the review team and while considering the sources of bias
- If two or more domains scored an unclear RoB, the overall score was unclear RoB
- If two or more domains scored a high RoB, the overall score was high RoB

Completed RoB templates are available on request.

**Risk of bias (RoB) in individual systematic reviews based on the ROBIS tool.**

| **Study** | **1. Study eligibility** | **2. Study selection** | **3. Data collection & study appraisal** | **4. Synthesis & findings** | **RoB in the review** | **Reasons for receiving an unclear or high risk of bias rating (number corresponds to ROBIS section)** |  |
| --- | --- | --- | --- | --- | --- | --- | --- |
| **Systematic reviews on unhealthy food and sugar-sweetened beverages n=31** | | | | | | | |
| Afshin 2017 (1) | L | L | L | L | L | - |  |
| Andreyeva 2022 (2) | L | L | L | L | L | - |  |
| Green 2013 (3) | L | L | L | L | L | - |  |
| Hammaker 2022 (4) | L | L | L | L | L | - |  |
| Lhachimi 2020 (5) | L | L | L | L | L | - |  |
| Pfinder 2020 (6) | L | L | L | L | L | - |  |
| Teng 2019 (7) | L | L | L | L | L | - |  |
| Mackenbach 2019 (8) | L | L | L | L | L | - |  |
| Mackenbach 2022 (9) | L | L | L | L | L | - |  |
| Nakhimovsky 2016 (10) | L | L | L | L | L | - |  |
| Von Philipsborn 2020 (11) | L | L | L | L | L | - |  |
| Backholer 2016 (12) | L | L | L | U | L | 4. Did not include variability around the reported estimates and no information about the robustness of findings. |  |
| Mizdrak 2015 (13) | L | L | U | L | L | 3. Unclear if steps were taken to minimise errors in the data collection or RoB assessments. |  |
| Nikniaz 2022 (14) | L | L | L | U | L | 4. No information was given about the robustness of findings, and it was unclear about how RoB assessments were used to interpret the findings. |  |
| Alagiyawanna 2015 (15) | L | U | L | U | U | 2. No information was given about the study selection procedure. The application of the English language filter and terms relating to English language were inconsistent and unclear.  4. Did not include information about between-study variability or the robustness of findings. |  |
| Niebylski 2015 (16) | L | L | U | U | U | 2. Lack of clarity about the data collection process, and whether efforts were made to minimise errors in the RoB assessment. Whether the RoB tool used was fit-for-purpose was unclear. |  |
| Andreyeva 2022 (17) | L | L | L | H | U | 4. There was large between-study variability which was not addressed or explored and no assessment of the impact of RoB scores on the results was made. |  |
| Engler-Stringer 2014 (18) | L | L | H | L | U | 3. Data collection process was not described, and no RoB assessment was undertaken. |  |
| Itria 2021 (19) | L | L | L | H | H | 4. Given the similarity in outcome measures, it is unclear why a meta-analysis was not undertaken and reporting of variability between studies and for study estimates was inconsistent. |  |
| Redondo 2018 (20) | L | L | L | H | H | 4. Unclear why a meta-analysis could not be undertaken given the similarity of outcomes. Lacked transparency about between-study variation or the robustness of findings. Did not incorporate their assessments of RoB within studies in their results. |  |
| Powell 2013 (21) | L | L | U | H | H | 3. Unclear whether steps were taken to reduce errors in the data collection process and whether RoB assessments were made.  4. Did not provide a funnel plot or sensitivity analysis to assess robustness of findings. RoB of studies within the review was not assessed and could not be considered. |  |
| Cabrera Escobar 2013 (22) | L | H | H | L | H | 2. Did not use methods additional to database searching to identify relevant reports. Did not give sufficient information about the terms and structure of the search strategy. Did not specify restrictions based on date, publication format, or language. Lack of clarity about study selection procedure.  3. Unclear whether data extraction was undertaken in duplicate, and no RoB assessment was undertaken. |  |
| Dodd 2020 (23) | L | L | H | H | H | 3. Sufficient detail on the population/sample/dataset the data was reported or not clear and measures of variability were not consistently reported.  4. There were deviations from the published protocol that were not explained and a lack of transparency about between-study variability and the robustness of findings. |  |
| Mah 2019 (24) | L | L | H | H | H | 3. No efforts were made to reduce errors in data collection processes which were undertaken by a single researcher without any checks. No RoB assessments were undertaken.  4. Given the similarity in outcome measures, it is unclear why results were not meta-analysed. Between-study variability was large, and not addressed, and no assessment of the robustness of findings was made. |  |
| Pérez-Ferrer 2019 (25) | L | H | H | U | H | 2. Did not use additional methods to database searching to identify relevant reports. Title-and-abstract screening was undertaken by a single researcher and it was unclear if full text screening was done in duplicate. The use of selected filters may have missed relevant hits.  3. No information was given about whether steps were taken to reduce errors in the data collection process, and no RoB assessments were undertaken.  4. It is unclear whether all eligible studies were included in the synthesis and no information was given about the robustness of the findings. |  |
| Holsten 2009 (26) | L | H | U | H | H | 2. Terms and structure of search strategy may not have retrieved as many eligible studies as possible. Lack of clarity about study selection process.  3. Data collection process was not described, and no information was given about how RoB assessments were made.  4. It was unclear whether all eligible studies were included and the approach to synthesis was unclear. No consideration of the influence of RoB in studies was made. |  |
| Thow 2010 (27) | L | H | H | H | H | 2. Did not provide enough information about the search strategy to understand if the terms and structure would retrieve as many eligible papers as possible. Information about the eligibility of studies based on date and publication format was unclear. No information given about study selection process.  3. Doesn’t describe the data collection process clearly enough to understand whether a template was used and what steps were taken to reduce error. Did not extract variability around estimates. It is unclear what the approach to assessing RoB was.  4. Unclear why a meta-analysis was not undertaken given the similarity of findings, and between-study variation was not adequately considered. Did not incorporate their assessments of RoB within studies in their results. |  |
| Powell 2009 (28) | L | H | H | H | H | 2. Did not use additional database searching to identify relevant reports. Did not provide a sample search strategy to understand if the terms and structure would retrieve as many eligible papers as possible.  3. Did not use formal data extraction templates and did not undertake RoB assessments.  4. Between-study variability was not assessed, and no information was given about the robustness of findings. RoB of studies within the review was not assessed and could not be considered. |  |
| Maniadakis 2013 (29) | H | L | H | H | H | 1. The eligibility criteria were ambiguous because it was not clear which countries or populations were included, or whether unpublished studies were sought.  3. No RoB assessment was undertaken.  4. The structure of the synthesis was unclear and there was no consideration about how RoB in studies might influence the results. |  |
| Thow 2014 (30) | H | U | U | H | H | 1. The eligibility criteria were ambiguous because it was not clear which countries or populations were included. The eligibility dates were limited, only including papers published between 2009-2012.  2. Did not use additional methods to database searching to identify relevant reports. No information given about study selection process.  3. Unclear whether steps were taken to reduce errors in data collection and RoB assessments.  4. Between-study variation was not explicitly dealt with, or discussed, and insufficient information was given to understand if the findings were robust. Did not incorporate their assessments of RoB within studies in their results. |  |
| Wright 2017 (31) | H | U | H | H | H | 1. Not enough information was given to assess whether the eligibility criteria were appropriate and unambiguous.  2. Unclear if study selection process was undertaken in duplicate. The eligibility criteria were unclear regarding which foods and types of tobacco were eligible. The lack of clarity was to some extent reflected in the search terms.  3. Unclear what steps were taken to reduce errors in data collection and although the authors mention a ‘matrix’, it is not clear what data were extracted. It is unclear whether all relevant results were collected for use in the synthesis and RoB assessments were not undertaken.  4. It is unclear whether all eligible studies were included in the synthesis. A lack of information was given to understand between-study variability and only direction of effect was reported. No information about the robustness of findings was given. No RoB assessment was undertaken to consider alongside the findings. |  |
| **Systematic reviews on tobacco=9** | | | | | | | |
| Akter 2023 (32) | L | L | L | L | L | - |  |
| Guindon 2015 (33) | L | L | L | L | L | - |  |
| Thomas 2008 (34) | L | L | L | L | L | - |  |
| Kjeld 2023 (35) | L | L | L | L | L | - |  |
| McKay 2015 (36) | L | L | L | L | L | - |  |
| Nazar 2021 (37) | L | L | L | H | U | 4. Unclear why a meta-analysis was not undertaken, and the between-study variability was not addressed or discussed. No information was provided about the robustness of findings. |  |
| Wilson 2012 (38) | L | L | L | H | U | 4. Between-study variability was not assessed, and RoB assessments were not considered in the results. |  |
| Hill 2014 (39) | L | L | U | H | H | 3. It’s unclear if all relevant results were collected for use in the synthesis and there was a lack of information about how RoB assessments were made, whether the tool was appropriate, and whether efforts to minimise error in these assessments were undertaken.  4. Between-study variability was not addressed or discussed, and it was unclear whether findings were robust. Assessments of RoB were not clearly incorporated into the results. |  |
| Jawad 2018 (40) | L | L | H | H | H | 3. Did not undertake RoB assessments.  4. The stated number of included studies doesn’t reflect the number of studies included in the meta-analysis and review. Between-study variability and robustness of findings was not assessed. No RoB assessment was undertaken to understand the influence of this on the results. |  |
| **Systematic reviews on alcohol n=9** | | | | | | | |
| Kilian 2023 (41) | L | L | L | L | L | - |  |
| Scott 2017 (42) | L | L | L | L | L | - |  |
| Elder 2010 (43) | L | L | U | L | L | 3. Data collection process not described and unclear whether efforts to minimise error in RoB assessments were made. |  |
| Wagenaar 2010 (44) | L | L | U | L | L | 3. Does not describe how data extraction was undertaken or the process of assessing RoB. |  |
| Kõlves 2020 (45) | L | L | U | L | L | 3. Does not include information about whether steps were taken to minimise errors in the data collection or RoB assessments. |  |
| Li 2015 (46) | U | U | U | U | U | 1. The eligibility criteria were not clearly described, and it was unclear whether unpublished studies were sought.  2. Did not provide enough information about the search strategy to understand if the terms and structure would retrieve as many papers as possible. No information given about the study selection process.  3. No information about efforts to minimise error in data collection processes or RoB assessments.  4. Lacked information about between-study variability and the robustness of findings. |  |
| Baldwin 2022 (47) | L | L | U | H | H | 3. Does not describe data collection processes or steps taken to minimise error in RoB assessments.  4. Unclear why a meta-analysis was not undertaken given the similarity of findings. Limited information given about the variability around study estimates and variability between studies. The influence of RoB assessments was not considered in the results. |  |
| Wilson 2014 (48) | H | L | H | H | H | 1. No pre-published protocol or clear rationale for review. The description about eligible outcomes was unclear. Alcohol sales or purchases seemed to be ineligible meaning the eligibility criteria were not appropriate for the review’s question.  3. Does not describe data extraction process and no RoB assessment was undertaken.  4. Seemed to include studies which should have been excluded according to their stated eligibility criteria. |  |
| Wagenaar 2009 (49) | L | U | H | H | H | 2. Lack of information about the date, publication format, and language restrictions. No information was given about the study selection process.  3. Data collection process not described, and no RoB assessments were undertaken.  4. Funnel plots were not provided, and sensitivity analyses were not undertaken. No RoB assessments were undertaken so the influence of bias could not be assessed. |  |
| **Systematic reviews on multiple commodities n=1** | | | | | | | |
| Miracolo 2021 (50) | L | L | L | H | U | 4. Between-study variation or robustness of findings was not assessed despite high variability. |  |

H = high risk of bias, L = low risk of bias, U = unclear; RoB: risk of bias

**Risk of bias across the included systematic reviews reporting on alcohol using the ROBIS tool.**

**Risk of bias across the included systematic reviews reporting on tobacco using the ROBIS tool.**

**Risk of bias across the included systematic reviews reporting on unhealthy food and sugar-sweetened beverages using the ROBIS tool.**

**Appendix 8: Overview of the findings of systematic reviews reporting the relationship between price and demand (excludes meta-analyses which are reported in Table 2). The number of estimates can be greater than the number of included studies if a study included more than one estimate. Reviews are ordered from low to high risk of bias, and within that, alphabetically.**

| **Study author (ref); search period** | **Included study designs; population** | **Intervention** | **Subgroup**  **(if applicable)** | **Range in PEDs or number of estimates with their direction of effect** | **ROBIS score** |
| --- | --- | --- | --- | --- | --- |
| **Systematic reviews on unhealthy food and/or soft drinks n=21** | | | | | |
| **Unhealthy food n=9** | | | | | |
| Lhachimi 2020 (5)  Inception – 2019 | Observational=2  All ages=2 | Saturated fat tax | All ages | Inverse association: 2/2 | L |
| Pfinder 2020 (6)  Inception – 2019 | Observational=1  All ages=1 | Sugar-containing food tax | All ages | Inverse association: 1/1 | L |
| Mackenbach 2019 (8)  Inception – 2018 | Observational=3  Adults=2, all ages=1 | Fast-food price/tax | Fast food | Inverse association: 2/2 | L |
| Mizdrak 2015 (13)  1980 – 2014 | Experimental=6  Adults=6 | High energy density/calorie food price/tax | Adults | Range= ns (NR) to -1.80 | L |
| Andreyeva 2022 (17)  Inception – 2020 | Observational=14  All ages=14 | Non-essential energy-dense food, saturated fat, candy, and snacks tax | Non-essential energy dense food and confectionary | Inverse association: 4/6  Mixed association: 1/6  No association: 1/6 | U |
|  |  |  | Candy and snacks | No association: 1/1 |  |
|  |  |  | Saturated fat | Inverse association: 2/2 |  |
| Engler-Stringer 2014 (18)  1995 – 2013 | Observational=1  Children=1 | Fast food price | Children | Inverse association: 1/1 | U |
| Dodd 2020 (23)  2000 – 2019 | Observational=4, experimental=2  All ages=5, adults=1 | High salt containing food tax | - | No association: 4/7  Inverse association: 3/7 | H |
| Mah 2019 (24)  Inception – 2018 | Observational=10, experimental=8  NR, all ages were eligible | Unhealthy food price/tax (NR) | - | Inverse association: 10/18  No association: 4/18  Mixed association: 4/18 ^[[1]](#footnote-2)^ | H |
| Thow 2014 (30)  2009 – 2012 | Observational=7, experimental=1  Adults=8 | High calorie for nutrient food tax | Adults | Inverse association: 5/7  No association: 2/7 | H |
| **Sugar-sweetened beverages n=7** | | | | | |
| Nakhimovsky 2016 (10)  1990 – 2016 | Observational=7  All ages=7 | SSB price/tax | All ages | Inverse association: 7/7 | L |
| Nikniaz 2022 (14)  Inception – 2021 | Experimental=4  All ages=2, adults=1, children=1 | SSB price/tax | - | Inverse association: 4/4 | L |
| Von Philipsborn 2020 (11)  Inception – 2018 | Observational=1  All ages=1 | SSB price/tax | All ages | Inverse association: 1/1 | L |
| Alagiyawanna 2015 (15)  Inception – 2013 | Observational=9  Children=4, adults=3, all ages=2 | SSB tax | Children | No association: 2/3  Inverse association: 1/3 | U |
|  |  |  | All ages | Inverse association: 1/1 |  |
| Itria 2021 (19)  2009 – 2019 | Observational=16  All ages=6, adults=6, children=4 | SSB price/tax | All ages | Inverse association: 8/8 | H |
|  |  |  | Adults | Inverse association: 7/7 |  |
|  |  |  | Children | Inverse association: 1/2  No association: 1/2 |  |
| Pérez-Ferrer  2019 (25)  1999 – 2017 | Observational=3  All ages=3 | SSB tax | All ages | Inverse association: 3/3 | H |
| Redondo 2018 (20)  2011 – 2017 | Observational=11, experimental=6  All ages=17 | SSB tax | All ages | Inverse association: 14/16  No association: 2/16 | H |
|  |  |  |  |  |  |
| **Unhealthy food and SSBs n=5** | | | | | |
| Niebylski 2015 (16)  2003 – 2013 | Observational=6, experimental=2  All ages=7, children=1 | SSB and unhealthy food (NR) price/tax | Unhealthy food | Inverse association: 1/1 | U |
|  |  |  | SSBs | Inverse association: 3/5  Positive association: 1/5  No association: 1/5 |  |
| Maniadakis 2013 (29)  1990 – 2013 | Observational=37, experimental=4  All ages=20, adults=11, children=10 | SSB and high fat food price/tax | SSB | Inverse association: 13/13 | H |
|  |  |  | High fat food | Inverse association: 5/6  Mixed association: 1/6 |  |
|  |  |  | SSB and high fat food combined | Inverse association: 2/2 |  |
| Powell 2013 (21)  2007 – 2012 | Observational=40  Children=21, adults=10, all ages=9 | SSB and fast-food price/tax | SSB | Range= -0.71 to -3.87 | H |
|  |  |  | Fast food | Range= -0.47 to -0.57 |  |
| Thow 2010 (27)  2000 – 2019 | Observational=6  NR, all ages were eligible | SSB and unhealthy food (NS) tax | - | Inverse association: 9/9 ^[[2]](#footnote-3)^ | H |
| Wright 2017 (31)  1990 – 2015 | Observational=8  NR, all ages were eligible | SSB and unhealthy food (NS) tax | - | Inverse association: 4/8  No/positive association^[[3]](#footnote-4)^: 4/8 | H |
| **Tobacco n=4** | | | | | |
| Kjeld 2023 (35)  2011 – 2021 | Observational=6  People <30 years=6 | Tobacco price/tax | Initiation  (people <30 years) | Range= -1.13 to -0.11 | L |
|  |  |  | Consumption  (people <30 years) | Range= -1.93 to -0.50 |  |
|  |  |  | Prevalence  (people <30 years) | Range= -0.69 to -0.13 |  |
|  |  |  | Cessation  (people <30 years) | Positive association: 1/1 |  |
| McKay 2015 (36)  Inception – 2013 | Observational=1  Adults=1 | Tobacco price/tax | Adults | Inverse association: 1/1 | L |
| Nazar 2021 (37)  Inception – 2020 | Observational=28  All ages=28 | Tobacco price/tax | All ages | Range = -0.02 to -0.88 | U |
| Wilson 2012 (38)  Inception – 2009 | Observational=35  Adults=16, children=12, all ages=7 | Tobacco price/tax | Prevalence (children) | Range= -0.14 to -0.01 | U |
|  |  |  | Initiation (children) | Range= -0.07 to -0.001 |  |
|  |  |  | Cessation (children) | Range= 0.038 to 0.12 |  |
|  |  |  | Prevalence (adults) | Inverse association: 12/16  No association: 4/16 |  |
|  |  |  | Cessation (adults) | Inverse association: 5/6  Mixed association: 1/6 |  |
| **Alcohol n=3** | | | | | |
| Elder 2010 (43)  Inception – 2005 | Observational=78  NR, all ages were eligible | Alcohol price/tax | All alcohol (aggregate studies) | Median= -0.77  (IQI= -2.00, -0.50) | L |
|  |  |  | All alcohol (individual studies) | Range= -0.29 to -3.54 |  |
|  |  |  | Spirits | Median= -0.79  (IQI = -0.90, -0.24) |  |
|  |  |  | Beer | Median= -0.50  (IQI -0.91, -0.36) |  |
|  |  |  | Wine | Median= -0.64  (IQI -1.03, -0.38) |  |
| Scott 2017 (42)  Inception – 2015 | Observational=1  Children=1 | Alcohol price/tax | Children | Range= -0.73 to -0.18 | L |
| Li 2015 (46)  1980 – 2013 | Observational=2  All ages=2 | Alcohol tax | All ages | Inverse association: 2/2 | U |
| **Multiple commodities n=1** | | | | | |
| Miracolo 2021 (50)  2000 – 2018 | Observational=14  All ages=14 | Unhealthy food (NR), SSB and alcohol price/tax | SSBs | Inverse association: 8/8 | H |
|  |  |  | Unhealthy foods | Inverse association: 2/2 |  |
|  |  |  | Tobacco | PED range= -0.28 to -0.87 |  |
|  |  |  | Alcohol | Inverse association: 1/1 |  |

Only data pertaining to our research question and eligibility criteria was extracted. For example, if a review undertook a meta-analysis of studies evaluating both the impact of a tobacco tax and a smoking ban, only information pertaining to tax was extracted. As such, the numbers of studies listed in this table might not match the numbers listed in the published review.

H: high risk of bias; IQI: inter-quartile interval; NR: not reported; L: low risk of bias; PED: price elasticity of demand; ROBIS: risk of bias in systematic reviews; SES: socioeconomic status; SSB: sugar-sweetened beverage; U: unclear risk of bias

**Appendix 9: Overview of the findings of systematic reviews and/or meta-analyses reporting the relationship between price and health outcomes. The number of estimates can be greater than the number of included studies if a study included more than one estimate. Reviews are ordered from low to high risk of bias, and within that, alphabetically.**

| **Study author (ref); search period** | **Included study designs; population** | **Intervention** | **Subgroup**  **(if applicable)** | **Pooled PED/range/n estimates with their direction of effect** | **ROBIS score** |
| --- | --- | --- | --- | --- | --- |
| **Systematic reviews on unhealthy food and/or soft drinks n=13** | | | | | |
| **Sugar-sweetened beverages n=6** | | | | | |
| Mackenbach 2022 (9)  Inception – 2021 | Observational=1  All ages=1 | SSB tax | Dental caries | Inverse association: 1/1 | L |
| Nakhimovsky 2016 (10)  1990 – 2016 | Observational=7  All ages=7 | SSB price/tax | BMI (all ages) | Inverse association: 4/4 | L |
| Hammaker 2022 (4)  2000 – 2022 | Observational=17  NR, all ages were eligible | SSB price/tax | Visits to dentists for dental caries | Inverse association: 1/1 | L |
| Alagiyawanna 2015 (15)  Inception – 2013 | Observational=9  Children=4, adults=3, all ages=2 | SSB tax | BMI (children) | No association: 2/2 | U |
|  |  |  | BMI (adults) | Inverse association: 2/4  No association: 2/4 |  |
|  |  |  | Excess weight (children) | No association: 4/5  Inverse association: 1/5 |  |
|  |  |  | Excess weight (adults) | Inverse association: 2/2 |  |
|  |  |  | Excess weight (all ages) | No association: 1/1 |  |
| Cabrera Escobar 2013 (22)  2000 – 2013 | Observational=12  All ages=8, adults=3, children=1 | SSB price/tax | Overweight (% points) | Range= -0.0002 to -0.045 | H |
|  |  |  | Obesity (% points) | Range= -0.0001 to -0.34 |  |
| Itria 2021 (19)  2009 – 2019 | Observational=16  All ages=6, adults=6, children=4 | SSB price/tax | Weight (all ages) | Inverse association: 2/3  No association: 1/3 | H |
|  |  |  | Weight (adults) | Inverse association: 3/5  No association: 2/5 |  |
|  |  |  | Weight (children) | Inverse association: 2/4  No association: 2/4 |  |
| **Unhealthy food and SSBs n=5** | | | | | |
| Niebylski 2015 (16)  2003 – 2013 | Observational=6, experimental=2  All ages=7, children=1 | SSB and unhealthy food (NR) price/tax | Weight | Inverse association: 2/4  No association: 1/4  Mixed association: 1/4 | U |
| Maniadakis 2013 (29)  1990 – 2013 | Observational=37, experimental=4  All ages=20, adults=11, children=10 | High fat food price/tax | Weight | Inverse association: 10/11  No association: 1/11 | H |
|  |  | SSB price/tax | Weight | Inverse association: 5/7  No association: 2/7 |  |
| Powell 2009 (28)  1990 – 2008 | Observational=7  Adults=5, children=2 | Fast food price/tax | BMI (children) | Inverse association: 3/3 | H |
|  |  |  | BMI (adults) | Inverse association: 7/10  No association: 3/10 |  |
|  |  | Sugar price/tax | BMI (adults) | Inverse association: 2/2 |  |
| Powell 2013 (21)  2007 – 2012 | Observational=40  Children=21, adults=10, all ages=9 | SSB price/tax | BMI/weight | Inverse association: 6/9  Positive/no association^[[4]](#footnote-5)^: 3/9 | H |
|  |  | Fast food price/tax | BMI/weight | Inverse association: 40/58  Positive/no association^[[5]](#footnote-6)^: 18/58 |  |
| Thow 2010 (27)  2000 – 2009 | Observational=6  NR, all ages were eligible | SSB and unhealthy food (NR) tax | Weight | Inverse association: 6/13  No association: 6/13  Positive association: 1/13 | H |
|  |  |  | Ischemic heart disease deaths | Inverse association: 1/1 |  |
|  |  |  | Cardiovascular disease deaths | Inverse association: 2/3  No association: 1/3 |  |
| **Unhealthy food n=2** | | | | | |
| Andreyeva 2022 (17)  Inception – 2020 | Observational=14  All ages=14 | Non-essential energy-dense food, saturated fat, candy and snacks tax | BMI (all ages) | No association: 2/2 | L |
| Holsten 2009 (26)  Inception – 2006 | Observational=1  Children=1 | Fast food price | BMI (children) | No association: 1/1 | H |
| **Alcohol n=6** | | | | | |
| Elder 2010 (43)  Inception – 2005 | Observational=78  NR, all ages were eligible=78 | Alcohol price/tax | Liver disease deaths | Inverse association: 4/5  No association: 1/5 | L |
|  |  |  | Alcohol-related cancer deaths | Inverse association: 1/1 |  |
|  |  |  | Deaths from suicide | Inverse association: 1/1 |  |
|  |  |  | Deaths from homicide, falls, fires/burns and other injuries | No association: 1/1 |  |
|  |  |  | Alcohol dependence | Inverse association: 1/1 |  |
|  |  |  | Sexually transmitted infections | Inverse association: 1/1 |  |
| Wagenaar 2010 (44)  Inception – 2009 | Observational=50  Adults=27, all ages=12, children=11 | Alcohol price/tax | Alcohol-related morbidity and mortality | Pooled PED= -0.35  (95% CI= -0.46, -0.23) | L |
|  |  |  | Other all-cause mortality and industrial injury | Pooled PED= -0.08  (95% CI= -0.15, 0.001) |  |
|  |  |  | Deaths from suicide | Pooled PED= -0.05  (95% CI= -0.10, 0.01) |  |
|  |  |  | Sexually transmitted infections | Pooled PED= -0.06  (95% CI= -0.08, -0.03) |  |
| Kõlves  2020 (45)  Inception – 2009 | Observational=8  All ages=7, adults=1 | Alcohol price/tax | Deaths from suicide (all sexes) | Inverse association: 4/6  No association: 2/6 | L |
|  |  |  | Deaths from suicide (men) | Positive association: 3/5  Inverse association: 2/5 |  |
|  |  |  | Deaths from suicide (women) | No association: 5/5 |  |
| Li 2015 (46)  1980 – 2013 | Observational=2  All ages=2 | Alcohol tax | Alcohol-related deaths | Inverse association: 4/4 | U |
|  |  |  | Cardiovascular disease deaths | Inverse association: 2/2 |  |
| Baldwin 2022 (47)  2010 – 2021 | Observational=2  Children=2 | Alcoholic RTD tax | Alcohol-related hospital admissions (children) | Inverse association: 3/4  No association: 1/4 | H |
| Wilson 2014 (48)  1992 – 2013 | Observational=2  All ages=2 | Alcohol price/tax | Deaths from homicide (women) | No association: 1/1 | H |
|  |  |  | Deaths from homicide (men) | No association: 1/1 |  |
| **Tobacco n=2** | | | | | |
| Akter 2023 (32)  Inception – 2021 | Observational=5  NR, all ages were eligible=5 | Tobacco price/tax | Lung cancer and respiratory symptoms and disease | Inverse association: 3/3 | L |
|  |  |  | Hospital admissions for cardiovascular disease | Inverse association: 1/1 |  |
|  |  |  | Hospital admission and discharge rates due to lung disease | Inverse association: 1/1 |  |
| Miracolo 2021 (50)  2000 – 2018 | Observational=14  All ages=14 | Tobacco price/tax^[[6]](#footnote-7)^ | Acute myocardial infarction | No association: 1/1 | H |

BMI: body mass index; CI: confidence interval; H: high risk of bias; L: low risk of bias; NR: not reported; PED: price elasticity of demand; ROBIS: risk of bias in systematic reviews; RTD SSB: sugar sweetened beverage; U: unclear risk of bias

**Appendix 10: Overview of the findings of systematic reviews reporting the relationship between price and demand or health outcomes by deprivation group. Reviews are ordered from those with a low to high risk of bias, and within that, alphabetically.**

| **Study author (ref); search period** | **Intervention** | **Subgroup**  **(if applicable)** | **Results of most deprived group (estimate n)** | **ROBIS score** |
| --- | --- | --- | --- | --- |
| Thomas 2008 (34)  Inception – 2006 | Tobacco price/tax | Low-income / occupation / education vs high (adults) | More responsive: 9/11  Less responsive: 1/11  No difference: 1/11 | L |
| Backholer 2016 (12)  Inception – 2015 | SSB tax | Low-income / education vs high | More responsive: 3/4  No difference: 1/4 | L |
| Mackenbach 2019 (8)  Inception – 2018 | SSB price/tax | Low-income vs high | More responsive: 1/1 | L |
| Miracolo 2021 (50)  2000 – 2018 | Unhealthy food (NR), SSB and alcohol price/tax | Low SES vs high | More responsive: 4/4 | L |
| Nazar 2021 (37)  Inception – 2020 | Tobacco price/tax | Low-income / SES vs high | More responsive: 1/1 | U |
| Dodd 2020 (23)  2000 – 2019 | High salt containing food tax | Low SES vs high | More responsive: 1/1 | H |
| Hill 2014 (39)  2006 – 2010 | Tobacco price/tax | Low-income / education group vs high (adults) | More responsive: 5/8  Less responsive: 2/8  No difference: 1/8 | H |

H: high risk of bias; L: low risk of bias; NR: not reported; ROBIS: risk of bias in systematic reviews; SES: socioeconomic status; SSB: sugar sweetened beverage; U: unclear risk of bias

**References used in appendices.**

1. Afshin A, Penalvo JL, Del Gobbo L, Silva J, Michaelson M, O'Flaherty M, et al. The prospective impact of food pricing on improving dietary consumption: a systematic review and meta-analysis. PloS one. 2017;12(3):e0172277.

2. Andreyeva T, Marple K, Marinello S, Moore TE, Powell LM. Outcomes following taxation of sugar-sweetened beverages: a systematic review and meta-analysis. JAMA Network Open. 2022;5(6):e2215276-e.

3. Green R, Cornelsen L, Dangour AD, Turner R, Shankar B, Mazzocchi M, et al. The effect of rising food prices on food consumption: systematic review with meta-regression. Bmj. 2013;346.

4. Hammaker J, Anda D, Kozakiewicz T, Bachina V, Berretta M, Shisler S, et al. Systematic review on fiscal policy interventions in nutrition. Frontiers in Nutrition. 2022;9:967494.

5. Lhachimi SK, Pega F, Heise TL, Fenton C, Gartlehner G, Griebler U, et al. Taxation of the fat content of foods for reducing their consumption and preventing obesity or other adverse health outcomes. Cochrane Database of Systematic Reviews. 2020(9).

6. Pfinder M, Heise TL, Boon MH, Pega F, Fenton C, Griebler U, et al. Taxation of unprocessed sugar or sugar‐added foods for reducing their consumption and preventing obesity or other adverse health outcomes. Cochrane Database of Systematic Reviews. 2020(4).

7. Teng AM, Jones AC, Mizdrak A, Signal L, Genç M, Wilson N. Impact of sugar‐sweetened beverage taxes on purchases and dietary intake: Systematic review and meta‐analysis. Obesity Reviews. 2019;20(9):1187-204.

8. Mackenbach JD, Nelissen KG, Dijkstra SC, Poelman MP, Daams JG, Leijssen JB, et al. A systematic review on socioeconomic differences in the association between the food environment and dietary behaviors. Nutrients. 2019;11(9):2215.

9. Mackenbach JD, Ibouanga EL, van der Veen MH, Ziesemer KA, Pinho MG. Relation between the food environment and oral health—systematic review. European journal of public health. 2022;32(4):606-16.

10. Nakhimovsky SS, Feigl AB, Avila C, O’Sullivan G, Macgregor-Skinner E, Spranca M. Taxes on sugar-sweetened beverages to reduce overweight and obesity in middle-income countries: a systematic review. PloS one. 2016;11(9):e0163358.

11. von Philipsborn P, Stratil JM, Burns J, Busert LK, Pfadenhauer LM, Polus S, et al. Environmental interventions to reduce the consumption of sugar‐sweetened beverages and their effects on health. Cochrane Database of Systematic Reviews. 2020(6).

12. Backholer K, Sarink D, Beauchamp A, Keating C, Loh V, Ball K, et al. The impact of a tax on sugar-sweetened beverages according to socio-economic position: a systematic review of the evidence. Public health nutrition. 2016;19(17):3070-84.

13. Mizdrak A, Scarborough P, Waterlander WE, Rayner M. Differential responses to food price changes by personal characteristic: a systematic review of experimental studies. PloS one. 2015;10(7):e0130320.

14. Nikniaz L, Tabrizi JS, Farhangi MA, Pourmoradian S, Allameh M, Hoseinifard H, et al. Community-based Interventions to Reduce Fat Intake in Healthy Populations: A Systematic Review and Meta-Analysis. Current Nutrition & Food Science. 2022;18(7):649-69.

15. Alagiyawanna A, Townsend N, Mytton O, Scarborough P, Roberts N, Rayner M. Studying the consumption and health outcomes of fiscal interventions (taxes and subsidies) on food and beverages in countries of different income classifications; a systematic review. BMC Public Health. 2015;15(1):1-14.

16. Niebylski ML, Redburn KA, Duhaney T, Campbell NR. Healthy food subsidies and unhealthy food taxation: A systematic review of the evidence. Nutrition. 2015;31(6):787-95.

17. Andreyeva T, Marple K, Moore TE, Powell LM. Evaluation of economic and health outcomes associated with food taxes and subsidies: a systematic review and meta-analysis. JAMA network open. 2022;5(6):e2214371-e.

18. Engler-Stringer R, Le H, Gerrard A, Muhajarine N. The community and consumer food environment and children’s diet: a systematic review. BMC public health. 2014;14(1):1-15.

19. Itria A, Borges SS, Rinaldi AEM, Nucci LB, Enes CC. Taxing sugar-sweetened beverages as a policy to reduce overweight and obesity in countries of different income classifications: a systematic review. Public health nutrition. 2021;24(16):5550-60.

20. Redondo M, Hernández-Aguado I, Lumbreras B. The impact of the tax on sweetened beverages: a systematic review. The American journal of clinical nutrition. 2018;108(3):548-63.

21. Powell LM, Chriqui JF, Khan T, Wada R, Chaloupka FJ. Assessing the potential effectiveness of food and beverage taxes and subsidies for improving public health: a systematic review of prices, demand and body weight outcomes. Obesity Reviews. 2013;14(2):110-28.

22. Cabrera Escobar MA, Veerman JL, Tollman SM, Bertram MY, Hofman KJ. Evidence that a tax on sugar sweetened beverages reduces the obesity rate: a meta-analysis. BMC public health. 2013;13:1-10.

23. Dodd R, Santos JA, Tan M, Campbell NR, Ni Mhurchu C, Cobb L, et al. Effectiveness and feasibility of taxing salt and foods high in sodium: a systematic review of the evidence. Advances in Nutrition. 2020;11(6):1616-30.

24. Mah CL, Luongo G, Hasdell R, Taylor NG, Lo BK. A systematic review of the effect of retail food environment interventions on diet and health with a focus on the enabling role of public policies. Current Nutrition Reports. 2019;8:411-28.

25. Pérez-Ferrer C, Auchincloss AH, de Menezes MC, Kroker-Lobos MF, de Oliveira Cardoso L, Barrientos-Gutierrez T. The food environment in Latin America: a systematic review with a focus on environments relevant to obesity and related chronic diseases. Public health nutrition. 2019;22(18):3447-64.

26. Holsten JE. Obesity and the community food environment: a systematic review. Public health nutrition. 2009;12(3):397-405.

27. Thow AM, Jan S, Leeder S, Swinburn B. The effect of fiscal policy on diet, obesity and chronic disease: a systematic review. Bulletin of the World Health Organization. 2010;88:609-14.

28. Powell LM, Chaloupka FJ. Food prices and obesity: evidence and policy implications for taxes and subsidies. The Milbank Quarterly. 2009;87(1):229-57.

29. Maniadakis N, Kapaki V, Damianidi L, Kourlaba G. A systematic review of the effectiveness of taxes on nonalcoholic beverages and high-in-fat foods as a means to prevent obesity trends. ClinicoEconomics and Outcomes Research. 2013:519-43.

30. Thow AM, Downs S, Jan S. A systematic review of the effectiveness of food taxes and subsidies to improve diets: understanding the recent evidence. Nutrition reviews. 2014;72(9):551-65.

31. Wright A, Smith KE, Hellowell M. Policy lessons from health taxes: a systematic review of empirical studies. BMC public health. 2017;17(1):1-14.

32. Akter S, Islam MR, Rahman MM, Rouyard T, Nsashiyi RS, Hossain F, et al. Evaluation of Population-Level Tobacco Control Interventions and Health Outcomes: A Systematic Review and Meta-Analysis. JAMA Network Open. 2023;6(7):e2322341-e.

33. Guindon GE, Paraje GR, Chaloupka FJ. The impact of prices and taxes on the use of tobacco products in Latin America and the Caribbean. American journal of public health. 2015;105(3):e9-e19.

34. Thomas S, Fayter D, Misso K, Ogilvie D, Petticrew M, Sowden A, et al. Population tobacco control interventions and their effects on social inequalities in smoking: systematic review. Tobacco control. 2008;17(4):230-7.

35. Kjeld SG, Jørgensen MB, Aundal M, Bast LS. Price elasticity of demand for cigarettes among youths in high-income countries: a systematic review. Scandinavian Journal of Public Health. 2023;51(1):35-43.

36. McKay AJ, Patel RK, Majeed A. Strategies for tobacco control in India: a systematic review. PLoS One. 2015;10(4):e0122610.

37. Nazar GP, Sharma N, Chugh A, Abdullah S, Lina S, Mdege ND, et al. Impact of tobacco price and taxation on affordability and consumption of tobacco products in the South-East Asia Region: A systematic review. Tobacco Induced Diseases. 2021;19.

38. Wilson LM, Avila Tang E, Chander G, Hutton HE, Odelola OA, Elf JL, et al. Impact of tobacco control interventions on smoking initiation, cessation, and prevalence: a systematic review. Journal of environmental and public health. 2012;2012.

39. Hill S, Amos A, Clifford D, Platt S. Impact of tobacco control interventions on socioeconomic inequalities in smoking: review of the evidence. Tobacco control. 2014;23(e2):e89-e97.

40. Jawad M, Lee JT, Glantz S, Millett C. Price elasticity of demand of non-cigarette tobacco products: a systematic review and meta-analysis. Tobacco control. 2018;27(6):689-95.

41. Kilian C, Lemp JM, Llamosas-Falcón L, Carr T, Ye Y, Kerr WC, et al. Reducing alcohol use through alcohol control policies in the general population and population subgroups: a systematic review and meta-analysis. Eclinicalmedicine. 2023.

42. Scott S, Muirhead C, Shucksmith J, Tyrrell R, Kaner E. Does industry-driven alcohol marketing influence adolescent drinking behaviour? A systematic review. Alcohol and alcoholism. 2017;52(1):84-94.

43. Elder RW, Lawrence B, Ferguson A, Naimi TS, Brewer RD, Chattopadhyay SK, et al. The effectiveness of tax policy interventions for reducing excessive alcohol consumption and related harms. American journal of preventive medicine. 2010;38(2):217-29.

44. Wagenaar AC, Tobler AL, Komro KA. Effects of alcohol tax and price policies on morbidity and mortality: a systematic review. American journal of public health. 2010;100(11):2270-8.

45. Kõlves K, Chitty KM, Wardhani R, Värnik A, De Leo D, Witt K. Impact of alcohol policies on suicidal behavior: a systematic literature review. International journal of environmental research and public health. 2020;17(19):7030.

46. Li Q, Babor TF, Zeigler D, Xuan Z, Morisky D, Hovell MF, et al. Health promotion interventions and policies addressing excessive alcohol use: a systematic review of national and global evidence as a guide to health‐care reform in China. Addiction. 2015;110:68-78.

47. Baldwin R, Miller PG, Coomber K, Patafio B, Scott D. A systematic narrative review of the effects of alcohol supply reduction policies on children and adolescents. International Journal of Drug Policy. 2022;101:103581.

48. Wilson IM, Graham K, Taft A. Alcohol interventions, alcohol policy and intimate partner violence: a systematic review. BMC public health. 2014;14:1-11.

49. Wagenaar AC, Salois MJ, Komro KA. Effects of beverage alcohol price and tax levels on drinking: a meta‐analysis of 1003 estimates from 112 studies. Addiction. 2009;104(2):179-90.

50. Miracolo A, Sophiea M, Mills M, Kanavos P. Sin taxes and their effect on consumption, revenue generation and health improvement: a systematic literature review in Latin America. Health Policy and Planning. 2021;36(5):790-810.

1. Includes estimates for the impact of subsidies on healthy food demand which could not be separated out. [↑](#footnote-ref-2)
2. Includes estimates for the impact of subsidising healthy foods which could not be separated out. [↑](#footnote-ref-3)
3. Not significant and positive associations could not be separated out. [↑](#footnote-ref-4)
4. Not significant and positive associations could not be separated out. [↑](#footnote-ref-5)
5. Not significant and positive associations could not be separated out. [↑](#footnote-ref-6)
6. Review reported on unhealthy food, SSB, tobacco and alcohol price/tax [↑](#footnote-ref-7)
